# Evaluating the potential of in-depth chrono-cultural and functional analysis of pottery in European cave archaeology: a case study from the prehistoric Grotte Di Sant’angelo Cave Complex (Cassano allo Ionio – Calabria, Italy)

**DOI:** 10.1007/s12520-025-02332-1

**Published:** 2026-01-24

**Authors:** Delia Carloni, Felice Larocca, Peter A. J. Attema, Giuseppe De Luca, Francesco Breglia, Marco Pacciarelli, Giuseppe E. De Benedetto

**Affiliations:** 1 Groningen Institute of Archaeology, University of Groningen, Groningen, Netherlands; 2 Università degli Studi di Bari Aldo Moro, Gruppo di ricerca speleo-archeologica, Bari, Italy; 3 Centro di Ricerca speleo-archeologica ‘Enzo dei Medici’, Roseto Capo Spulico, Italy; 4 Laboratorio di spettrometria di massa analitica ed isotopica, Dipartimento di Beni Culturali, Università del Salento, Lecce, Italy; 5 Dipartimento di Geoscienze, Università degli Studi di Padova, Padova, Italy; 6 Dipartimento di Studi Umanistici, Università degli Studi di Napoli Federico II, Napoli, Italy

**Keywords:** Cave archaeology, Pottery analysis, Residue analysis, Prehistory, Copper Age, Contextual approach

## Abstract

This paper evaluates the potential of in-depth chronological and functional analysis of prehistoric pottery (4th millennium BCE) from the Grotte di Sant’Angelo Cave Complex, located in the municipality of Cassano allo Ionio (Calabria, Southern Italy). The underground system, formed by sulfuric acid speleogenesis, features a floor scattered with depressions, holes, and fractures of different depths hosting archaeological materials. A number of these contexts in the so-called ‘Trivio’ area, excavated in 2017 and reported on here, provide an excellent opportunity to pioneer a combined archaeological and scientific contextual approach to unravelling the use of pottery in the Grotte di Sant’Angelo Cave Complex through time. The approach chosen first establishes the chronology of pots through typological comparisons with sites offering key stratigraphic sequences for prehistoric material culture present in the Italian peninsula and islands. Next, a functional study is presented that focusses on the use-related morphological properties of the pots, their performance characteristics, and preserved organic residues. Based on presentation and evaluation of the resulting data, the authors then proceed to propose hypotheses on the use and meaning of the various pottery shapes present in individual contexts from the Trivio zone cave floor contexts. At a more general level, the aim of the authors is to show how a contextual approach, combining several research tools for pottery analysis can make an important contribution to the toolbox of scholars working in European cave archaeology, thereby increasing the discipline’s potential of resolving theoretically informed questions about human-cave entanglements in later prehistory.

**Supplementary Information:**

The online version contains supplementary material available at 10.1007/s12520-025-02332-1.

## Introduction

**Fig. 1:**
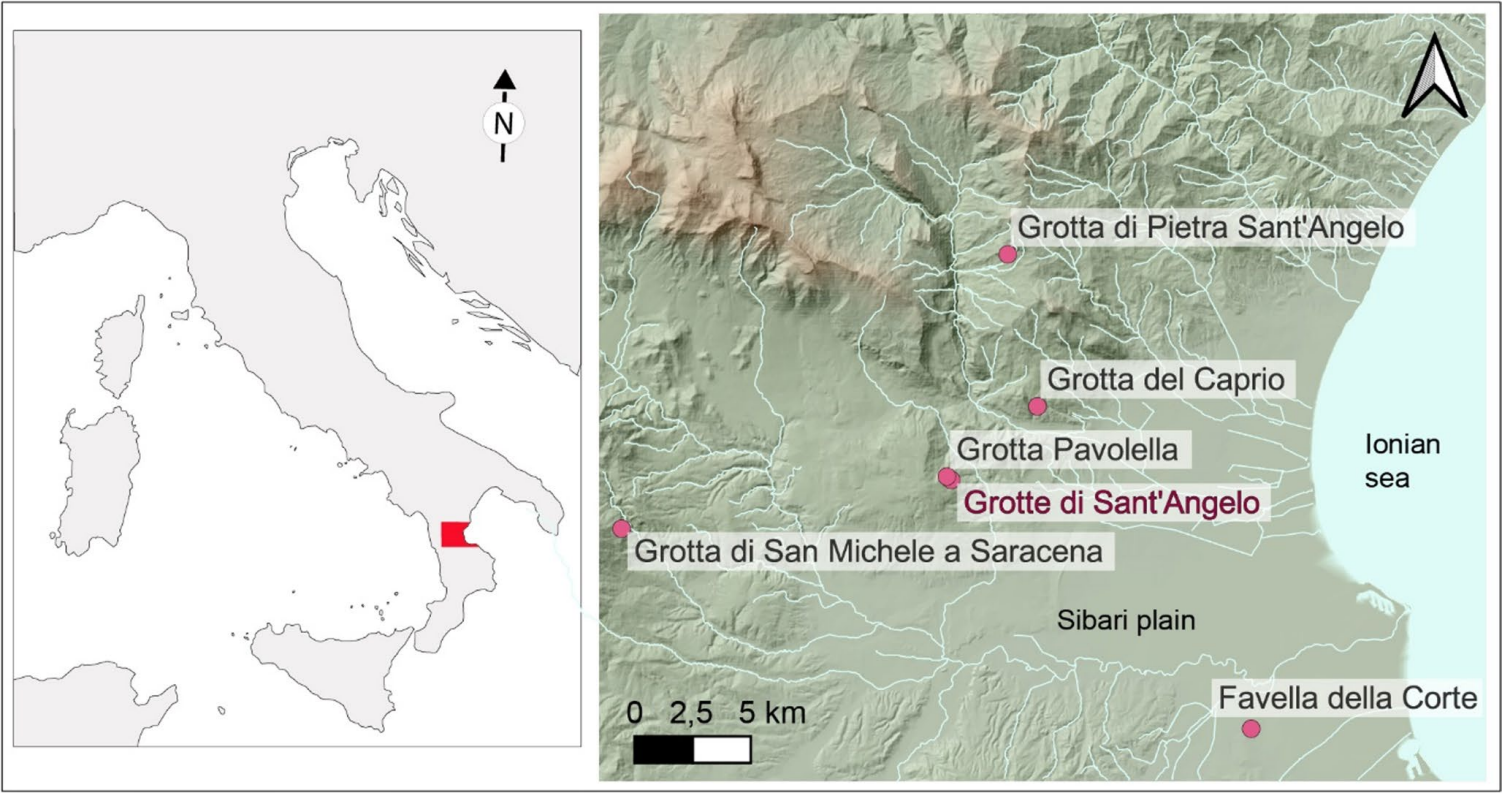
Location of the Grotte di Sant’Angelo Cave Complex with reference to the main Neolithic and Copper Age sites of the region. Map adapted from data provided by Tarquini et al. (2007)

Since the early 1990 s, cave archaeology has significantly advanced through the adoption of a contextual approach, the use of an increasingly diverse range of research methods, and the overcoming of a de-personalized vision of cave spaces (Bergsvik and Skeates 2012a). By identifying main contextual dimensions of relevance and considering the physical, sensory, and emotional sensations experienced by humans while entering in natural cavities (Bergsvik and Skeates 2012b; Dowd 2015; Dowd and Hensey 2016; Skeates 2016; López and Skeates 2022), researchers now fully explore the intimate human-cave relationship(s) (Straus 1990, 1997; Skeates 1994; Bonsall and Tolan-Smith 1997; Bergsvik and Skeates 2012b; Prijatelj and Skeates 2019). Scholars have observed that European caves and caverns were frequented by humankind to fulfill needs of both the practical and religious spheres (Bonsall and Tolan-Smith 1997; Angelucci et al. 2009; Dowd 2015; Agnolin 2021; Ustinova 2009) with the performance of religious-related rituals resulting from higher levels of symbolic engagement (Insoll 2004; Renfrew 2007; Bergsvik and Skeates 2012b; Clottes 2012; Dowd 2015). Furthermore, evidence suggests that in Europe the involvement with the darkness was outstandingly developed in Prehistoric times (Milisauskas 2011; Bergsvik and Skeates 2012b; Clottes 2012; Moyes 2012b; Büster et al. 2019). In particular, the so-called ‘dark zones’ (Faulkner 1988) often hosted Prehistoric ritual performances, stimulated and fostered by the peculiar features of these places: deep darkness, silence, temperature stability, intricate networks of passages and chambers, which led to intensified sensory experiences (Watson 2001; Lewis-Williams 2002; Ustinova 2009; Moyes 2012a; Dowd 2015; Skeates 2016; Prijatelj and Skeates 2019). Among the archaeological materials found in caves during the entire Post-Paleolithic Prehistory in Europe, pottery holds particular significance. From the Neolithic period onward (6th/5th millennium BCE), ceramic containers were frequently used in underground activities, including ritual offerings (Whitehouse 1992; Bonsall and Tolan-Smith 1997; Grifoni Cremonesi 2007; Milisauskas 2011; Bergsvik and Skeates 2012b; Moyes 2012b; Dowd 2015; Dowd and Hensey 2016; Büster et al. 2019). While chrono-cultural and functional analyses have been performed on ceramic finds from caves, their application largely remained confined to the theoretical frameworks of culture-historical and cognitive-processual approaches, offering valuable, but somewhat limited, information on the kind of ceramic containers discarded in caves and, in general, on the activities conducted in the underground spaces (e.g. Boschian and Montagnari-Kokelj 2000; Tomkins 2009; Benzi 2011; Šoberl et al. 2014; Tanasi 2014; Debels et al. 2020; Minelli and Guglielmi 2020; Pennetta et al. 2020b; Francés-Negro et al. 2021; Vykukal et al. 2021). A more interpretative, contextual, approach – one that considers the perceptions and experiences of past people – has been shown to provide deeper insights (Bergsvik and Skeates 2012a; Skeates 2016; Prijatelj and Skeates 2019; López and Skeates 2022). This work adopts such a perspective, looking at pottery shapes, decorations, morpho-functional properties, use-alteration traces and organic residues through a contextual lens. Specifically, we use pottery chrono-cultural and functional analysis to explore the six contextual dimensions of relevance to caves defined by Bergsvik and Skeates (2012a): the ‘architectural’, the stratigraphic and preservation, the spatial, the temporal, the scholarly, and the socio-economic contexts. The selected case study is the Grotte di Sant’Angelo Cave Complex^1^ (Cassano allo Ionio, Italy; Fig. 1), a sulfuric hypogenic cave frequented in prehistoric times (Tiné 1964; Larocca 2017) and presenting a peculiar physicality *sensu* Prijatelj and Skeates (2019). The archaeological material, primarily pots, were found inside holes and openings at the floor of natural, non-anthropogenic origin and were suspected to be related with ritual behavior (Larocca 2017). With this case study we test and evaluate the potential of in-depth chrono-cultural and functional analysis of pottery in European cave archaeology when adopting a contextual approach. Finally, this paper contributes to the development of the interdisciplinary approach to cave archaeology advocated by Prijatelj and Skeates (2019), which draws upon scientific techniques to address theoretically informed issues.

## Archaeological background

**Fig. 2:**
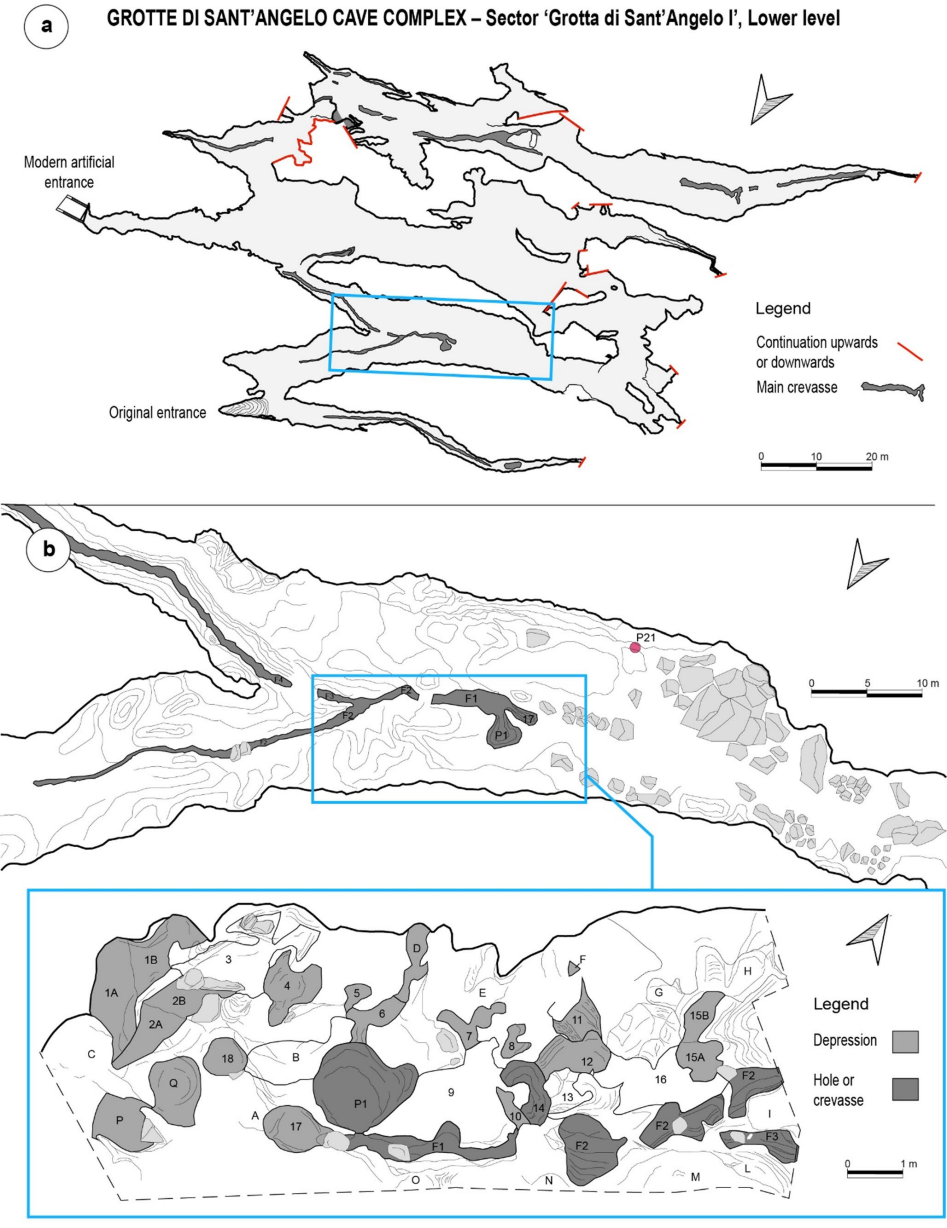
The archaeological context of the Grotte di Sant’Angelo Cave Complex: (**a**) Plan of the lower level of the cave, where the Trivio is located (light blue rectangle); (**b**) Detailed plan of the Trivio area and of the findspots of archaeological material (original drawings by Francesco La Carbonara, Davide Servidio and Ilaria De Marco, digitation and modifications by D. Carloni). Fracture F2 was investigated only in the Trivio area

The underground system of Grotte di Sant’Angelo developed for 3649 m into the dolomitic and calcareous Meso-Cenozoic successions composing the Monte San Marco hill, in the territory of the municipality of Cassano allo Ionio, in Calabria region, Italy (Fig. 1). The archaeological significance of the cave complex was first assessed by surveys and excavations conducted by Santo Tiné in the 1960s (Tiné 1962, 1964) who defined three sectors ‘I’, ‘II’, and ‘III’ based on main natural entrances (altitudes between 435 and 455 m a.s.l.). Tiné first focused on the sector named ‘Grotta di Sant’Angelo III’ and carried out excavation campaigns in 1962 and 1963. The archaeological deposits of this underground area testify to human presence during the Middle-Late Neolithic (layer IV, 5500–4000 BCE), Early Copper Age (layer III, 3700/3600–3300 BCE), Late Copper Age-Early-Bronze Age (layer II, 2350 − 2150 BCE), and Late Bronze Age (layer I, 1200–1100 BCE). Thanks to archaeological surveys and speleological explorations conducted in the sector named ‘Grotta di Sant’Angelo II’ it was also possible to recover Middle-Late Neolithic (5500–4000 BCE), Late Copper Age-Early-Bronze Age (2350 − 2150 BCE) and Middle Bronze Age (1650 − 1300 BCE) potsherds, lithic tools and a set of human remains associated with Early Bronze Age (2150–1650 BCE) pots and a bronze bracelet (Tiné 1964, 1987; Gasparo 1979, 1980; Ippolito 2017). The archaeological significance of the sector ‘Grotta di Sant’Angelo I’ – the largest one – was never ascertained before 2017 when the Centro Regionale di Speleologia “Enzo dei Medici” conducted a preliminary archaeological survey in the cave and found evidence of human presence in the so-called ‘Trivio’ area (Fig. 2). This work focuses on this latter sector of the Grotte di Sant’Angelo Cave Complex.

**Fig. 3:**
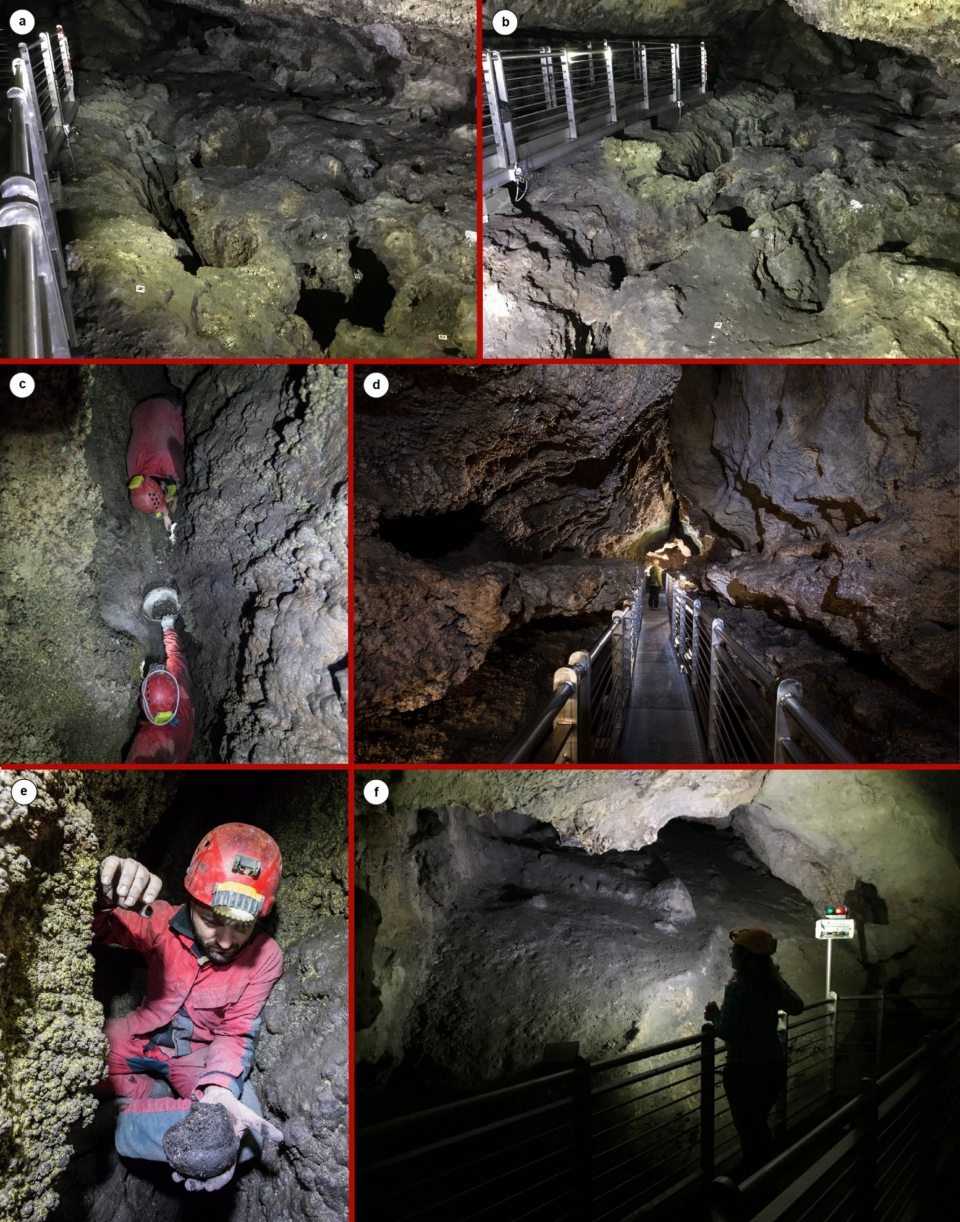
The Grotte di Sant’Angelo Cave Complex: (**a**) and (**b**) depressions and holes in the bedrock floor of the Trivio area (photo by Felice Larocca); (**c**) and **e**) difficult operations of recovering of archaeological artefacts in the ‘F2’ cavity (photo by Francesco De Salve); (**d**) typical morphology of caves passages marking the origin from sulfuric acid speleogenetic process (photo by Francesco De Salve); **f**) view from the inside of the original descending entrance to the cave, now obliterated by a sediment detrital cone (photo by Felice Larocca)

The Trivio is a cave room pervaded by deep darkness that serves as a junction between three diverging subterranean sectors (Larocca 2017). Archaeological finds were found in depressions and holes in the bedrock floor and in openings running longitudinally to the major axis of the cave space (Figs. 2 and 3) (Larocca 2017). These cavities are natural, non-anthropogenic, and resulted from the speleogenetic process that governed the formation of the underground complex, i.e. the sulfuric acid speleogenesis (Gasparo 1980; Galdenzi 1997; De Waele et al. 2016; Galdenzi and Menichetti 2017; Galdenzi and Maruoka 2019). As a matter of fact, the Grotte di Sant’Angelo Cave Complex formed above the water table by abiotic and/or biotic oxidation of H_2_S in rising hypogenic thermal water (De Waele et al. 2016). The relict sub-parallel, horizontal, and rectilinear passages, composing the underground complex and arranged in overlapping levels, were fed by inclined or vertical feeders, or directly from narrow slots in the floor (Galdenzi and Menichetti 2017; Galdenzi and Maruoka 2019). The holes and the openings where the archaeological material was found represent the remnant of the feeders, system of fissures and crevasses which formed during the sulfuric acid speleogenetic process (Galdenzi and Maruoka 2019). Notwithstanding their natural origin, these cave floor cavities became the object of suspected cultic activity likely involving the abandonment of pots, faunal remains, querns, and lithic industry inside them (Larocca 2017). The most important opening on the cave floor is the so-called ‘F2’ cavity, which runs along the major longitudinal axis of the Trivio area (Figs. 2 and 3) and represents a relic of a feeder/crevasse. Archaeological remains found in the holes on the ground were only slightly or partly covered by sediments due to the very low sediment production rate that accompanies the sulfuric acid speleogenetic process and features the caves resulted from (Klimchouk et al. 2017). Only in F2 cavity was it possible to distinguish three vertically stacked layers of sediment: US 1, US 2, and US 3. The first two levels encountered, US 1 and US 2, were ephemeral with US 1 composed of a slight veil of sandy gypsum deposit and US 2 of a brown terrigenous sediment. The third layer US 3 was brownish to blackish in color and silty to clayey in composition, very rich in charcoal and malacofauna. This level contained most of the archaeological material found in F2 and represents the most important sub-context of the Trivio area. The rest of the cavities on the cave floor did not undergo by significant sediment infillings and the archaeological material they hosted remained almost exposed at the surface.

## Materials and methods

**Fig. 4:**
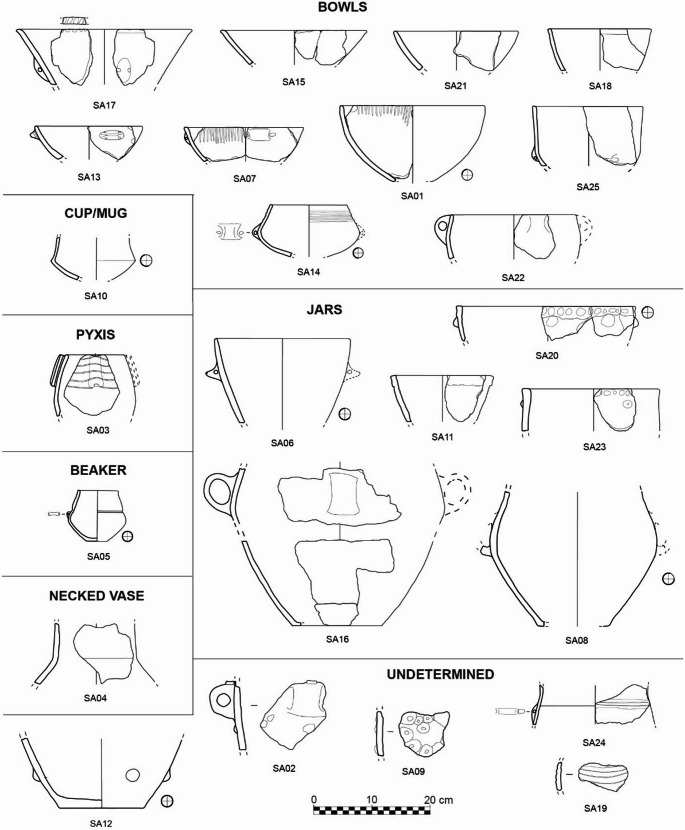
The representative set of 25 pots/potsherds from the archaeological survey conducted in 2017 in the Trivio area by the Centro Regionale di Speleologia “Enzo dei Medici”

The study of the ceramic assemblage from the Trivio area of the Grotte di Sant’Angelo Cave Complex was conducted at the Archaeological Deposit located in San Lorenzo Bellizzi (Calabria, Italy) thanks to the authorization of the Soprintendenza Archeologia, Belle Arti e Paesaggio per la Provincia di Cosenza. The detailed functional analysis concerned a representative set of 25 identified pots, selected based on their conservation status and informative potential. Table 1 contains the list of the samples with their shape characteristics and findspots in the Trivio area. Most of the pots were found in layer US 3 documented at the findspot F2 (Figs. 2 and 3) and US 1 from different natural cavities in the Trivio area. The set accounts for ten bowls, one cup/mug, six jars, a pyxis, a beaker, a necked vase, and five sherds whose original shape remains undetermined^2^ (Fig. 4, detailed information in Supplementary Information 1). In absence of a relevant sediment covering of the findings, and thus of a relevant stratigraphic sequence, the chronological framework of analyzed artefacts was assessed considering their stylistic traits – overall morphology, decoration, presence and types of suspension features and handle – and the occurrence of such attributes in the pottery traditions of Southern Italy. The morpho-functional characteristics of pots – such as capacity, weight, stability, accessibility, and transportability – were assessed through vessel shape-related metrical properties, following the guidelines by Orton et al. (1993), Skibo (2013), and Rice (2015), to explore their possible past uses. Similarly, internal and external surface’s treatments were used to infer on permeability, abrasion resistance, and heating and cooling effectiveness (Schiffer 1988, 1990; Young and Stone 1990; Schiffer et al. 1994; Pierce 2005; Skibo and Schiffer 2008; Harry et al. 2009; Skibo 2013; Ionescu and Hoeck 2020). A systematic observation of the traces left by the usage on the ceramic surfaces was carried out to unveil the function(s) the vessels performed. Distinction between signs related to the manufacturing process and use-alteration traces was done based on the parameters set by Schiffer and Skibo (1989), Skibo (1992, 2013, 2015), Skibo and Schiffer (2008), Forte et al. (2018), and Debels et al. (2020).

**Table 1:** List of pots/potsherds with their shapes, findspots in the Trivio area and indication of the part sampled for residue analysis

**POTS**	**SHAPE**	**FINDSPOT**	**SAMPLED AREA **(S1, S2)
SA01	Tronco-ellipsoid bowl, unrestricted	F2, US3	Lower interior side
SA02	Unknown	F2, US3	Interior side
SA03	Ovaloid pyxis, restricted	F2, US1	Mid interior side
SA04	Necked vase	F2, US3	Upper interior side
SA05	Biconical beaker	F2, US3	Mid interior side
SA06	Tronco-ovoidal jar, unrestricted	F2, US3	Upper interior side
SA07	Tronco-ellipsoid bowl, unrestricted	F2, US3	Mid interior side
SA08	Cordiform jar	F2, US3	Mid interior side
SA09	Unknown	F2, US3	Interior side
SA10	Carinated cup/mug, restricted	F2, US3	Lower interior side
SA11	Tronco-conical jar, unrestricted	F2, US3	Mid interior side
SA12	Unknown	F2, US3	Interior base
SA13	Tronco-ellipsoid bowl, unrestricted	F2, US3	Mid interior side
SA14	Ellipsoid bowl with a tronco-conical neck, restricted	F2, US3	Mid interior side
SA15	Tronco-conical bowl, unrestricted	F2, US3	Mid-lower interior side
SA16	Cordiform jar	F2, US3	Upper-mid interior side
SA17	Tronco-conical bowl, unrestricted	9, US1	Upper-mid interior side
SA18	Tronco-ellipsoid bowl, unrestricted	P, US1	Lower interior side
SA19	Unknown	P, US1	Interior side
SA20	Tronco-ovoidal jar, unrestricted	P, Q, US1	Upper interior side
SA21	Tronco-ellipsoid bowl, unrestricted	3, US1	Lower interior side
SA22	Tronco-ovoidal bowl, unrestricted	F3, US1	Mid interior side
SA23	Cylindrical jar, unrestricted	F4, US1	Upper interior side
SA24	Unknown, restricted	P21, US1	Interior side
SA25	Tronco-ellipsoid bowl, unrestricted	14, US1	Lower interior side

The analysis of the residues trapped in ceramic surfaces was executed at the University of Salento by means of High Temperature Gas Chromatography-Mass Spectrometry (HTGC-MS) and Gas Chromatography/Combustion/Isotope Ratio Mass Spectrometry (GC-C-IRMS). The investigation regarded the entire set of 25 pots/potsherds selected for this study. These were still covered by sediment, stored in plastic bags and did not receive any manipulation prior to sampling. The selection of samples was therefore done when the ceramics had not yet been washed, i.e. just as they appeared from the original depositional contexts, before cleaning operations started. To minimize contamination from recent human contact, sterilized gloves cleaned with alcohol were worn at all stages. The sampling area on the vessel was selected considering the shapes, functional characteristics, and observed use-alteration traces (Table 1). A hand drill served for sampling ceramic powder from the pots’ interior at progressive depth levels, distinguishing between a superficial layer up to a maximum depth of 1 mm (S1, ~ 0.1 g) and a deeper one encompassing the vessel’s section up to about half the thickness of its walls (S2, ~ 1 g). The samples were gathered on aluminium sheets and then transferred in glass vials: both metal sheets and all used glassware were previously cleaned at 450 °C for 12 h to minimize contamination. To identify residues linked to the burial context and potential contaminants from the deposition environment, 11 sediments from the depositional context of the pots were selected for analysis as well. These represented the stratigraphic sequence of F2 (US 1, US 2, and US 3) and infillings of cavities named 3, 9, 14, F3, F4, P, and P21 (Fig. 2).

Preparation of samples for GC-MS involved the addition of a 10 µl of internal standard (1 mg/ml tetratriacontane in hexane) to the ceramic powders and the sediments and solvent extraction of lipids following established protocols (Mottram et al. 1999; Debono Spiteri et al. 2016; Reber 2022). Total Lipid Extract (TLE) was obtained by adding 5 ml of Dichloromethane-Methanol (2:1, v: v) to the powdered samples and, after mixing and vortex agitation, extraction was carried out in an ultrasonic bath for 15 min (three times). Extracts were separated by centrifugation (3000 rpm, 10 min), collected and concentrated to about 1 ml in a centrifugal evaporator at 35 °C. Then, 500 µl of TLE were dried, added with a derivatization internal standard (20 µl of 0.01 mg/ml tridecanoic acid in hexane) and derivatized with 40 µl of the derivatizing agent N, O-bis(trimethylsilyl)trifluoroacetamide (BSTFA) at 70 °C for 60 min. A subset of ceramic samples was further treated with BF₃/butanol for identifying presence of fermentation products (Patrizi et al. 2025). Sherds were selected based on their morpho-functional characteristics and the presence of visible use-alteration traces potentially linked to fermentation products. In brief, the treatment involved the bowls SA01 and SA25, the jars SA04, SA08 and SA16, the cup/mug SA10, the pyxis SA03, the beaker SA05, and the base SA12 and consisted in the ceramic pellets subjected to 2–3 ml of butylating reagent (80 °C for 12 h). Thereafter, butyl esters were extracted with dichloromethane, subsequently dried, and derivatized with BSTFA to yield TMS derivatives of hydroxyl groups. All samples were analyzed in splitless mode using the Agilent 7890B/5977A Series Gas Chromatograph/Mass Selective Detector. The separation was carried out on a VF5HT column (Agilent, 30 m, 0.32 μm, 0.1 μm) using the following oven temperature program: measurement conditions: 60 °C (2 min), 10 °C/min up to 350 °C (20 min). The obtained chromatograms were analysed using Agilent’s ChemStation software. Peak identification was performed by comparison with the NIST mass spectra database. The total lipid concentration or TLE (µg/g) for each analysed sample was calculated based on the sum of the areas of all chromatogram peaks (Total Ion Current, TIC).

Compound Specific Isotope Analysis was performed on the S2 interior surface layer of vessel samples. After addition of 5 µg of tetratriacontane and 0.30 µg of tridecanoic acid, samples were methylated using methanol acidified with sulfuric acid, at 70 °C overnight, then methyl esters were extracted with hexane (3 ml for three times), and evaporated to about 0.2 ml then analysed by GC-C-IRMS. An Agilent Technologies 7890 A gas chromatograph was hyphenated to a Isoprime 100 stable isotopes mass spectrometer using the Isoprime GC5 interface [patrizi, 2024]. Methyl esters were separated on a HP1 column (50 m, id 0.320 mm, film thickness 0.17 μm) with the following temperature program: 80 °C (2 min), increased 5 °C min⁻¹ up to 300 °C (14 min). The He gas (99.9995% purity) was used as carrier gas with a flow of 1 mL min⁻¹. The injection was performed in split mode 1: 2 into split/splitless injector at 250 °C. The combustion tube, packed with CuO, operated at 850 ° C, while the gas chromatograph-furnace interface at a temperature of 350 °C. The ionization energy of the mass spectrometer was set at 80 eV, acquiring isotopes 44, 45 and 46 The certificated reference materials used for the calibration were n-undecane (δ13C: −26.11‰, Chiron C0414.11–150-CY) and n-pentadecane (δ13C: −30.22‰, Chiron C0418.15–150-CY). Each sample was analyzed three times, and the results were referred against the VPDB standard (Vienna-PDB, *Bellemnitella americana*; McCarroll and Loader 2004).

## Results

### Chronology of pots

**Fig. 5:**
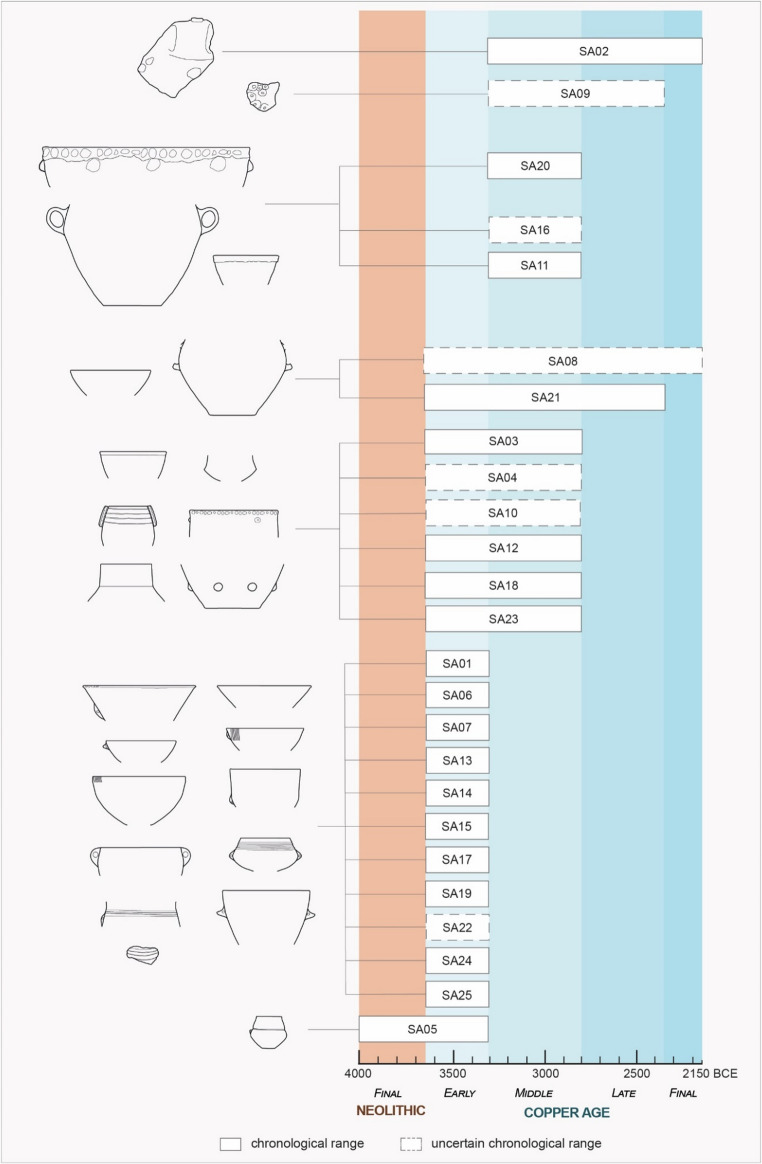
Chronology of pots/potsherds from the Trivio area based on typological comparisons

**Fig. 6:**
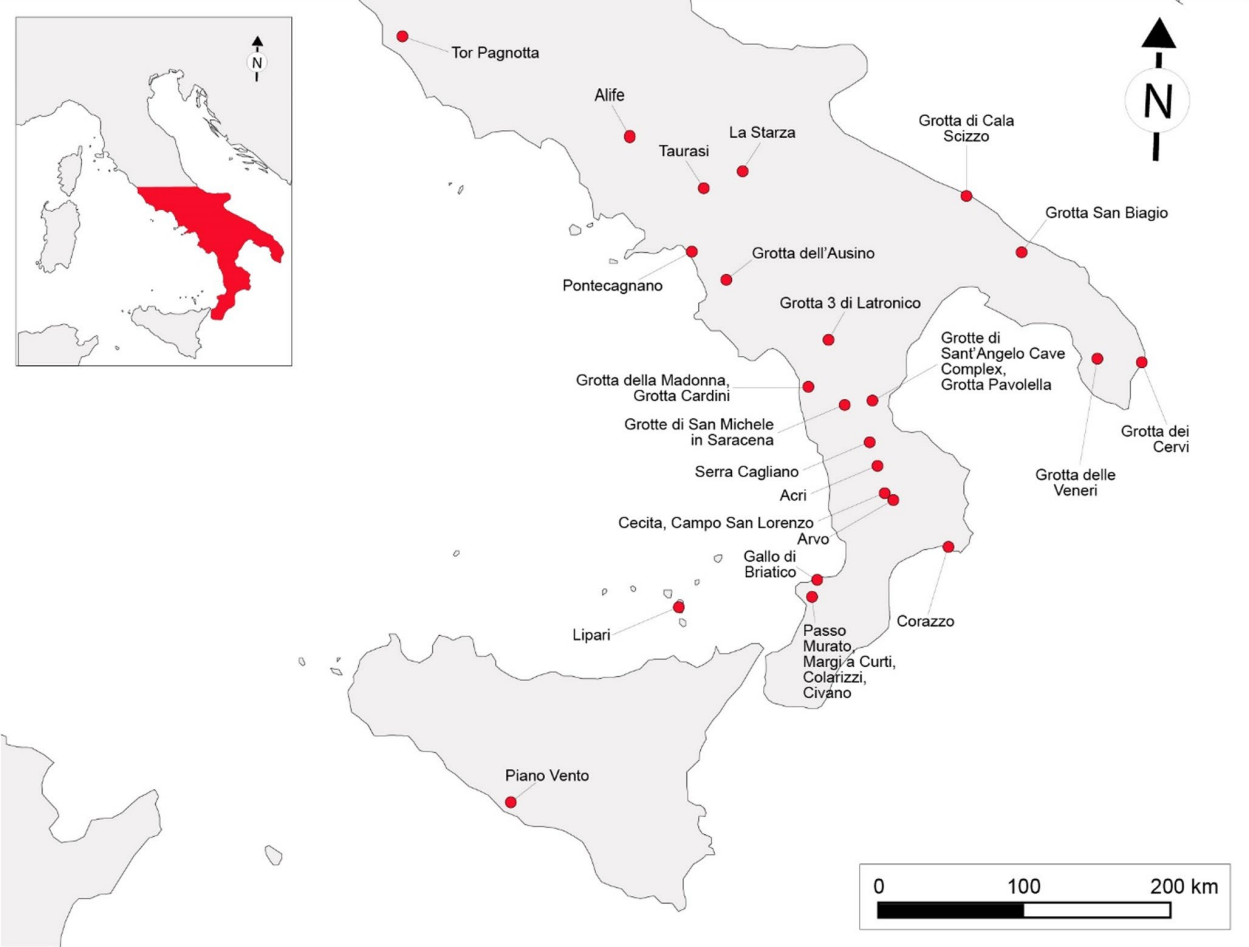
Location of the sites showing pottery with stylistic traits similar to the ceramic assemblage from the Trivio area of the Grotte di Sant’Angelo Cave Complex

The ceramic assemblage found in the Trivio area (Fig. 4) matches the stylistic characteristics of the prehistoric pottery material cultures of Southern and Central Italy, which allows direct comparisons with Copper Age sites (Supplementary Information 1) and enables the assessment of pot chronology (Fig. 5). The location of the sites cited herein is shown in Fig. 6.

The most ancient vessel is the biconical beaker SA05. It finds parallels with numerous Final Neolithic (4000 − 3650 BCE) to Early Copper Age (3650 − 3300 BCE) sites across various regions: Grotta Pavolella and Corazzo in Calabria (Guerzoni 2004; Nicoletti 2004); Grotta delle Veneri, Grotta di Cala Scizzo and Grotta dei Cervi in Puglia (Geniola and Tunzi 1980; Bernabò Brea and Revedin 2004; Cocchi Genick 2009); Piano Vento in Sicily (Cocchi Genick 2009); and Tor Pagnotta in Lazio (Anzidei and Carboni 2020). Most of the unrestricted bowls (SA01, SA07, SA13, SA15, SA17, SA25), the restricted bowl SA14, and the body fragments SA19 and SA24 can be confidently ascribed to the Early Copper Age thanks to shape characteristics as well as to the occurrence of parallel decorative grooves and the presence of subcutaneous handles that served for pots’ suspension. Indeed, these stylistic aspects are widely diffused in the Calabria region during the first half of the 4th millennium BCE and the beginning of the second half as attested by various sites: Grotta di Sant’Angelo III (layer III, Tiné 1964), Grotta Pavolella (Guerzoni 2004), Grotta di San Michele in Saracena (Natali et al. 2021), Grotta della Madonna (Bernabò Brea and Cavalier 2000), and Serra Cagliano 1 (Guerzoni and Amodio 2011) in Northern Calabria; Margi a Curti in the Tropea Promontory area (Pacciarelli 2011); Piano di Cecita and Paliati in the Sila massif (Marino and Nicoletti 2023). Furthermore, the subcutaneous suspension features and the decoration of the rim and internal surfaces of open shapes are typical for the Early Copper Age findings of the Aeolian Islands (Bernabò Brea and Cavalier 1960, 1980). Other shapes confidently assignable to the Early Copper Age are the jar SA06 which can be compared with the repertoires of Grotta Pavolella and Gallo di Briatico (Grandinetti et al. 2004; Guerzoni 2004) and the bowl SA22 which is similar to examples from Lago Arvo (Biddittu et al. 2004) and Taurasi (Talamo 2008a) sites.

The remaining pot types show characteristics that parallel with the production of multiple phases of the Copper Age. The bowl SA18, the jar SA23, and base SA12 can be dated from Early (3650 − 3300 BCE) to Middle Copper Age (3300 − 2800 BCE) thanks to similarities with the assemblages from Grotta di Sant’Angelo III (layer III, Tiné 1964), the Calabrian sites of Grotta Pavolella, Grotta di San Michele a Saracena, Gallo di Briatico, Colarizzi, Passo Murato, and Piano di Cecita (Grandinetti et al. 2004; Guerzoni 2004; Tiné and Natali 2004; Pacciarelli 2011) as well as the Grotta San Biagio context in the Puglia region (Coppola et al. 2011). Moreover, the pyxis SA03 can also be assigned to an Early to Middle Copper Age time span as it shows parallels with sites in Calabria – Grotta Pavolella and Serra Cagliano 1 – and in the Campania region: Grotta dell’Ausino, La Starza, and Alife (Guerzoni 2004; Talamo 2008b; Aurino et al. 2023). The carination of the cup/mug SA10 finds parallels with specimens from Grotta di Sant’Angelo III (layer III, Tiné 1964), Pontecagnano (Bailo Modesti and Salerno 1998) and Lipari Acropolis (Bernabò Brea and Cavalier 1980). The necked vase SA04 resembles examples from Grotta Pavolella, Passo Murato, Gallo di Briatico and Colarizzi and various types of the Gaudo material culture (Bailo Modesti and Salerno 1998; Grandinetti et al. 2004; Guerzoni 2004; Pacciarelli 2011). Finally, seven ceramic vessels exhibit stylistic features that hinder precise dating but are generally compatible with the Middle Copper Age. The bowl SA21 corresponds to similar shapes from Grotta di Sant’Angelo III (layer III, Tiné 1964), Grotta Pavolella, Passo Murato, Margi a Curti, and Gallo di Briatico (Grandinetti et al. 2004; Guerzoni 2004; Pacciarelli 2011), whereas the small jar SA11 corresponds to examples from Colarizzi, Gallo di Briatico, and Pontecagnano (Bailo Modesti and Salerno 1998; Pacciarelli 2011). Similarly, pots comparable to the jars SA08 and SA16 were previously found both in cave and open-air contexts from Calabria – Grotta Pavolella, Grotta San Michele di Saracena, and Campo San Lorenzo – and the Puglia region at Grotta San Biagio (Guerzoni 2004; Tiné and Natali 2004; Coppola et al. 2011; Marino and Nicoletti 2023). The jar SA20 finds parallels with the assemblages of Passo di Murato and Pontecagnano (Bailo Modesti and Salerno 1998; Pacciarelli 2011). The multiple buttons applied on the body sherds SA02 and SA09 correspond to examples from different parts of Italy (see details in Supplementary Information 1), but notably from Colarizzi, Acri Colle Dogna, Civano, and Grotta n. 3 di Latronico in Calabria and Basilicata (Cremonesi 1976; Ingravallo 2002; Castagna and Schiappelli 2004; Grandinetti et al. 2004; Pacciarelli 2011).

Despite the presence of vessels that correspond to multiple phases of the Copper Age, none of these show morphological characteristics or decorations that can be exclusively attributed to the Late and Final Copper Age, and none of the distinctive traits typical of these periods were identified. Stratigraphic inferences can be drawn for F2 cavity only (Sect. Archaeological background): the Early Copper Age pyxis SA03 from US 1 has the same chronological framework as most of the pots from the underlying US 2 and US 3. In conclusion, the ceramic assemblage from the Trivio area covers the entire 4th millennium BCE, encompassing the Final Neolithic, Early Copper Age, and Middle Copper Age.

### Morpho-functional characteristics and visible use-alteration traces

The morpho-functional characteristics and surfaces of the pottery found in the Trivio area of the Grotte di Sant’Angelo Cave Complex were examined in detail (Supplementary Information 1) to assess the use-related properties of the vessels, their performance characteristics, and any evidence of actual use (Tables 2 and 3). All information was used to infer pottery function.

**Table 2:** Performance matrix based on morphological features and use-alteration traces for the bowls, the cup/mug, the pyxis, the beaker, and the necked vase from the Trivio area

		**Bowls**										**Cup/Mug**	**Pyxis**	**Beaker**	**Necked vase**
		**SA01**	**SA07**	**SA13**	**SA14**	**SA15**	**SA17**	**SA18**	**SA21**	**SA22**	**SA25**	**SA10**	**SA03**	**SA05**	**SA04**
**Performance**	Capacity	3.5 l	>1.3 l	>0.9	>1.3	>1.7	>3.7	>1.2	>1.4	>2.6	>2.4	>0.8	>0.9	>0.4	>1.7
	Weight	>0.9 kg	>0.3 kg	>0.4	>0.5	>0.5	>1.3	>0.3	>0.5	>0.9	>0.5	>0.2	>0.6	0.3	>0.6
	Stability	High	High	High	High	High	High	High	High	Medium	High	High or Medium	Medium	High	Medium
	Accessibility	High	High	High	Medium	High	High	High	High	High	High	Medium	Low	Medium	Low
	Transportability	High	High	High	High	High	High	High	High	Medium	High	High	High	High	Medium
	Containment security	Low	Medium	Medium	High	Medium	Medium	Medium	Low	Low	Medium	ND	High	Medium	High
	Impermeability	Medium	Medium	Low	Low	Low	Low	Low	Medium	Low	Low	Low	Low	Low	Medium
**Use-alteration**	Discolorations	E, B, I	I	/	E	/	E, I	I	/	ND	/	/	I	/	I
	Soot patches	E, B	/	I	E, B, I	E?	E	/	/	ND	E, I	/	/	/	/
	Oxidation	E	/	E	E, B	ND	E	E	E	ND	/	E	E	/	/
	Spall detachments	E, R, B	/	E, I	E, I	ND	E	E	E, I	E	E, I	E, I	E	E, I	/
	Fractures	I	E	E	E	/	/	/	/	ND	/	E	/	/	E, I
	Cracks	/	I	/	/	/	/	/	I	ND	I	/	/	/	/
	Depressions	B	/	I	I	/	E	/	/	ND	/	I	/	/	I
	Pits	B	/	I	I	/	/	/	/	ND	/	I	/	/	I
	Striations	E	E	/	/	E	E	E	/	ND	/	/	/	/	/
	Scratches	E, R, I	/	/	E	/	/	I	/	ND	/	/	/	/	/

Levels of development of significant use-related properties: High, Medium, Low, No Evidence (/) or Not Determinable (ND)

Position of the use-alteration traces: Interior (I), Exterior (E), Rim (R), Base (B), No Evidence (/) or Not Determinable (ND)

**Table 3:** Performance matrix based on morphological features and use-alteration traces for the jars and the sherds of undetermined shapes from the Trivio area

		**Jars**						**Undetermined**				
		**SA06**	**SA11**	**SA20**	**SA23**	**SA08**	**SA16**	**SA02**	**SA09**	**SA12**	**SA19**	**SA24**
** Performance**	Capacity	> 3.6	> 1.2	> 3.7	> 2.7	> 9.7	> 18.6	/	/	> 3.5	/	> 2.2
	Weight	> 1.1	> 0.4	> 0.8	> 0.8	> 2.5	> 3	/	/	> 1.8	/	> 0.3
	Stability	Medium	High	High	Medium	Medium	Low	/	/	/	/	High
	Accessibility	High	High	High	High	Medium	Medium	/	/	Medium?	/	Medium
	Transportability	High	High	Medium	Medium	Medium to Low	Medium to Low	Medium?	/	Medium?	/	High
	Containment security	Medium	Medium	Medium	Medium	Medium	Medium	/	/	/	/	High?
	Impermeability	Medium	Low	Low	Low	Medium	Medium	Low	Low	Low	Low	Low
** Use-alteration**	Discolorations	E	I	/	/	E	E	/	/	I	E	E
	Soot patches	E, I	/	I, R	E	E	E	/	/	/	/	/
	Oxidation	E	/	E	/	E	E	/	/	/	/	/
	Spall detachments	E, I	E	E, I	E, I, R	/	/	E	E	E	I	E, I
	Fractures	I	E	/	/	E, I	E, I	I	I	E, I	/	E, I
	Cracks	E, I	/	E, I	E, I	/	/	/	/	/	/	E
	Depressions	/	I, R	I	I	E, I	E, I	E	E, I	E, I	/	/
	Pits	/	E, I	E	I	E, I	E, I	E, I	E, I	E, I	I	E
	Striations	E, I	E	E	E	/	/	/	/	/	/	/
	Scratches	E, I	/	/	/	E	E	E	/	/	/	/

Levels of development of significant use-related properties: High, Medium, Low, No Evidence (/) or Not Determinable (ND)

Position of the use-alteration traces: Interior (I), Exterior (E), Rim (R), Base (B), No Evidence (/) or Not Determinable (ND)

#### Bowls

The bowls mostly have similar **use-related properties and performance characteristics** (Fig. 4; Table 2): high stability ensured by a low center of gravity, high transportability due to light weight, and medium to low impermeability due to smoothed/burnished surfaces. Accessibility of the contents depends on the orientation of the vessel walls and is high for all unrestricted pots, with content that can be reached by hands or tools, and medium for the restricted bowl SA14 which only allows for the insertion of a tool. Transportation of liquid or semi-liquid content is hampered by the risk of spillage, except for SA14, whereas pouring is favored by lip orientation in pots SA07, SA15, SA17, and SA18, but hindered in bowls SA14, SA21, and SA22. All the bowls could be carried by hand, but thermal hazards were common when filled with hot content, a challenge that could have been mitigated by the possibility of suspending certain vessels (SA07, SA13, SA17, SA14, SA22, SA25). In the case of non-hot content, bowl SA17 may have needed to be carried by being embraced and secured against the chest considering its significant volume (diameter ~ 29.2 cm and capacity > 3.7 l). The morphological characteristics of the pots always allowed the content to be secured with a lid or, additionally, with a cloth/skin cover for vessels SA07, SA13, SA14, SA15, SA17, SA18, and SA25, which could be tied to a suspension feature when available. In addition to the characteristics already listed for all the bowls, the restricted and inflected shape of SA14 presents morphological characteristics that help retain the contents, particularly liquids, and retards evaporation during heating, thereby saving fuel during cooking. In addition, its smooth contours and thin walls provide excellent thermal conductivity and resistance to thermal stress.

**Fig. 7:**
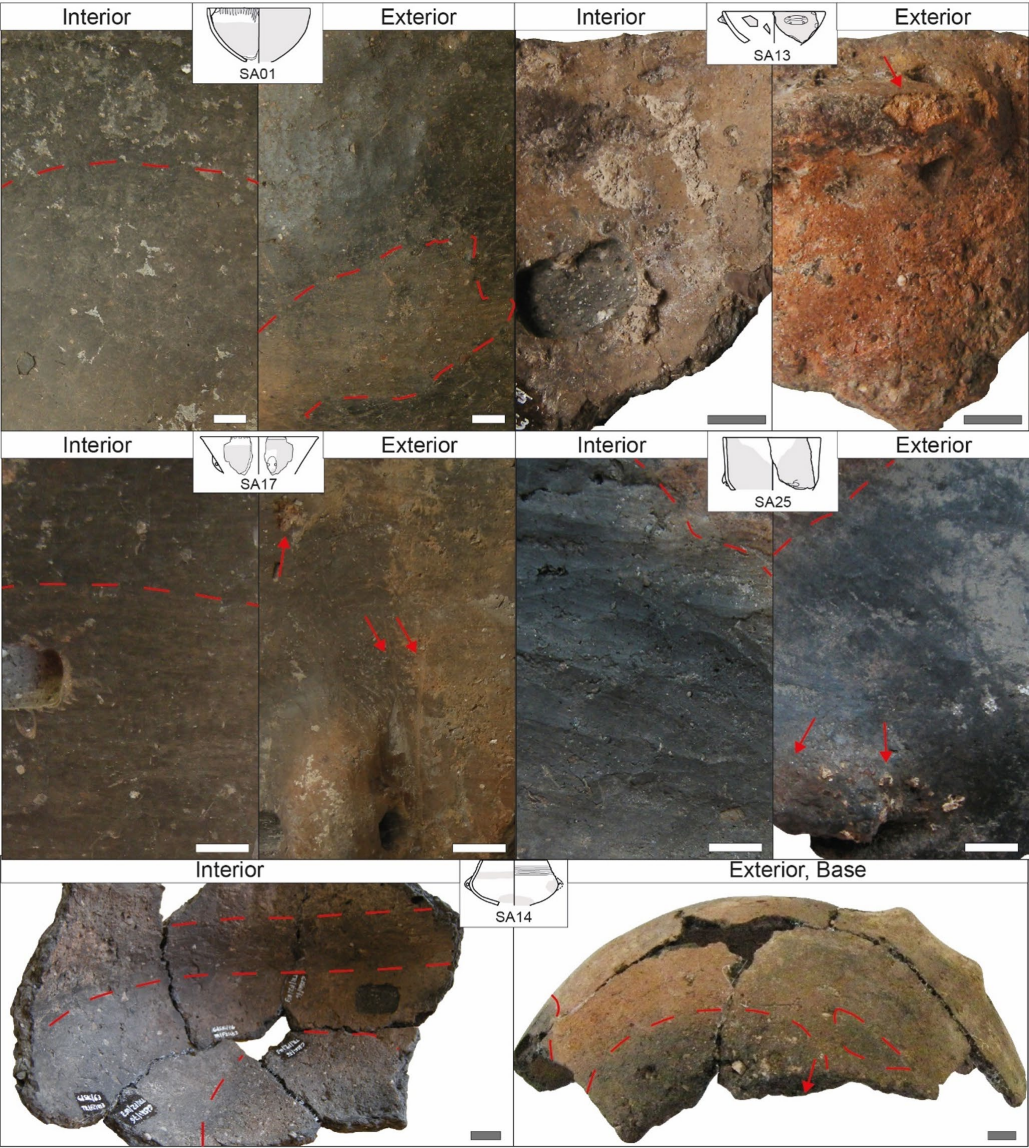
Exemplary use-alteration traces documented on bowls from Trivio area. The area interested by extensive traces is marked in gray in the drawing of the pot. Squared area devoid of material in pots SA13, SA14, and SA17 resulted from sampling operations for residue analysis. The length of the white/gray bar is always 1 cm

All bowls except SA07 and SA15 display signs of **alteration** due to the contact with fire such as discolorations, soot patches, oxidized areas, and spall detachments on the external surface (Table 2) eventually covering the areas of the suspension feature (Fig. 7). The interior also shows damage caused by heating such as incipient cracks and spall detachments (Table 2). Thermal alterations are particularly evident for SA01 (Fig. 7), causing glossy black patches and opaque oxidized areas on the exterior and a base rich in spall detachments, depressions, and pits. The exterior of SA13 became almost fully oxidized and severely damaged because of thermal shocks (Fig. 7). A particular case is represented by SA25 where glossy soot patches are visible in the same area both on the exterior and interior, reaching the rim area (Fig. 7). Furthermore, certain bowls with interiors that were not significantly affected by heating (SA01, SA07, SA17, SA18) show horizontal discoloration indicating the original level(s) of their content. This is evident in SA01 and SA17 (Fig. 7), where dyschromia clearly follows a horizontal pattern relative to the height of the vessel. Regarding abrasive wear, the bowls tend to show spall detachments and scratches on the external surfaces and on the rim as well (Table 2). In addition, striations and grooves created by a cord can be observed below the rim (SA07, SA15, SA18) or next to the suspension feature (SA17, Fig. 7). Finally, the bowl SA13 shows evidence of corrosive wear on the internal surface, which is characterized by spall detachments, depressions and pits leading to small areas of pedestalling inclusions (Fig. 7). A particular case is represented by the restricted bowl SA14 where the heating process led to the formation of a horizontal carbonized band in the upper interior part of the pot and a circular concentric band on the base area (Fig. 7). Depressions and pits on the interior of SA14 as well as extensive areas of pedestalling inclusions are possibly related to corrosive wear. Abrasive wear is indicated by a few scratches on the exterior. Concerning SA22, the calcareous encrustations covering the surfaces hinder any observation on the signs of use. A spall detachment is visible on the handle, but it cannot be determined whether this is related to fatigue wear or thermal shock.

The performance characteristics of bowls and their use-alteration traces are in line with the **function of meal preparation**,** serving and consumption**. Based on overall morphology, all pots may have been utilized to consume substances in a solid and (semi-)liquid state (Rice 1987; Skibo 2013). Consumption of soups or beverages is strongly supported by the vessels showing horizontal dyschromia on the internal surface, i.e. SA01, SA07, SA17, SA18, and for the bowl SA13 displaying evidence of corrosive wear (Reber 2022). Meal preparation presumably involved **heating** for all pots except SA07 and SA15, for which the available fragments do not show evidence of contact with fire. With regard to SA14, multiple lines of evidence suggest it was **suspended over a fire** and used for **wet cooking operations**, and served as a fundamental tool in **food processing** and **meal preparation**. Cooked substances could have corroded the pot’s interior.

#### Jars

The jars display variable **morpho-functional features** (Fig. 4; Table 3), which determine high to medium accessibility of their contents. Their stability ranges from medium to low, depending on the position of the center of gravity and, in some cases, the handles. Transportability also varies from low to high, depending on maximum diameter, size, weight, and capacity. The pots SA08 and SA16 have a medium to low transportability, which decreases depending on the content type (dry/semi-liquid/liquid) and weight, given their volumes of more than 9 and 18 l, respectively. In addition to being carried with the hands, SA06, SA20, and SA23 may have been held between the arms and secured against the chest. SA06 could also have been moved using cords. The size of SA08 and SA16 still allows them to be carried using the handles and against the body holding it between arms and against the chest, but the weight when filled may have required two people to move the vessel. The content could be presumably accessed by both hands and tools. Handling and transporting all jars when filled with liquid and/or hot contents may have been problematic due to risk of spillage and thermal hazards. The content could have been secured for all jars with a lid – possibly resting on flat lips in SA11, SA20, and SA23 – or with cloth/skin cover to be tied to the holes in the lugs (SA06), under the listel (SA11, SA20) or simply under the rim (SA23). Unfortunately, for SA08 and SA16, the containment security cannot be thoroughly assessed; it can only be noted that there appears to be a tendency towards a restricted opening. Impermeability is estimated as low to medium considering that the walls are generally thick except for SA20 and SA16 and the surfaces are smoothed for SA06 and SA11 and regularized for SA08, SA16, SA20 and SA23.

**Fig. 8:**
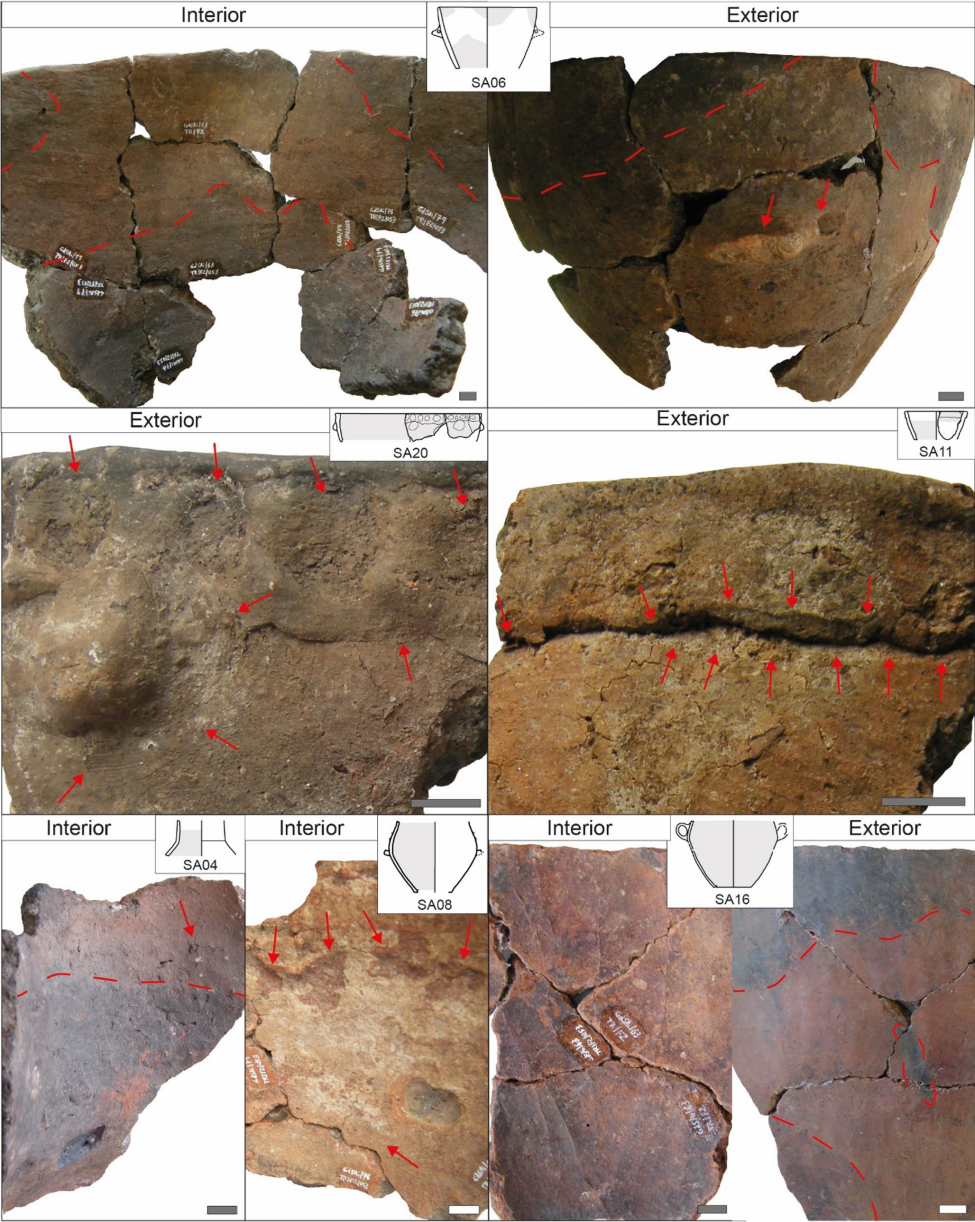
Exemplary use-alteration traces documented on jars from Trivio area. The area interested by extensive traces is marked in gray in the drawing of the pot. Squared area devoid of material in pots SA04 and SA08 resulted from sampling operations for residue analysis. The length of the white/gray bar is always 1 cm

**Use-alteration traces** vary between the different jars with evidence of different patterns of thermal stress for SA06, SA20 and SA23, of fatigue and abrasive wear for SA11, and various kinds of alterations for SA08 and SA16 (Table 3; Fig. 8).

The **tronco-ovoidal jar SA06** exhibits glossy black patterns of sooting on the internal surface, particularly on the lower part of the vessel toward the wall/base articulation (Fig. 8). These patches extend at certain points to the mid and upper parts of the vessel in the form of slightly vertical bands. Internal sooting is mirrored on the same area of the exterior by oxidation, whereas on the upper part of the vessel opaque black soot deposits and discoloration to gray are visible. Other evidence of thermal shock is cracks and few spall detachments occurring both on the internal and external surfaces and fractures visible on the interior. The lug is damaged by spall detachments that obliterated the original ending point of the prehension element and by incipient fractures near its holes (Fig. 8). Scratches are present on both the interior and exterior. Use-related alterations, along with performance characteristics, highlight the use of jar SA06 for **dry cooking operations** aimed at **toasting** foodstuffs (e.g. cereals or nuts) or **roasting** (Skibo 2013; Forte et al. 2018). Therefore, SA06 performed functions involved in **food processing and meal preparation**.

The **jars SA20 and SA23** show similar alterations formed during use, primarily related to thermal stress. Black soot covers the exterior of SA23 and the rim and internal surface of SA20, whereas oxidation occurs on the external surface of SA23 only. Cracks and spall detachments occur both on the interior and exterior, also covering the rim and the button areas in SA23. The finger impressions are highly ruined by grooves, spall detachments and pits in both vessels as well as the button of SA23 which exhibits spall detachments (Fig. 8). Abrasive and fatigue wear are indicated by striations visible in a circular pattern under the buttons (Fig. 8) and under the listel in SA20. Leveling can be seen on different spots on the rim and on the listel of SA20, whereas depressions and pits are present on the internal surface of SA23. Considering the pots’ roughened upper exterior, the performance characteristics and the thermal alterations, it can be assumed that SA20 and SA23 were used for **cooking**, possibly under wet conditions, although there is insufficient evidence to confirm this with certainty (Rice 1987; Skibo 2013, 2015; Forte et al. 2018).

Finally, the **jar SA11** exhibits signs of fatigue and abrasive wear primarily consisting of spall detachments on the external surface, fractures and pits on both the interior and the exterior, as well as depressions on the internal surface and the rim. In addition, striations and leveling are visible under the listel, likely marking the passage of a cord (Fig. 8). The internal surface shows horizontal discoloration indicating the original level(s) of the content during use. The overall shape and functional properties align with uses such as **serving or consuming** edibles/beverages; **storage** could also have been a function, though the amount of provisions would have been limited due to the pot’s modest capacity.

The **jars SA08 and SA16** show use-related alterations linked to both corrosive and thermal wear (Table 3; Fig. 8). The former consists of depressions, pits, and incipient fractures especially developed on the internal surface (Fig. 8) but present on the external one as well, which display limited areas of pedestalling inclusions (SA08; Supplementary Information 1). The latter includes a combination of sparse opaque soot deposits and oxidized, eventually discoloured, areas on the exterior (Fig. 8). The pot’s areas affected by thermal stress are traversed by fractures that may have favoured the pot to break into pieces. Concerning internal surfaces of these jars, they are almost all covered by a layer of residue, yellowish to whitish in SA08, and blackish in SA16, both strongly associated with evidence of corrosive processes for both jars and in association with spall detachment for SA16 only. Whereas residue-rich areas tend to be homogeneously distributed along the entire vessel’s interior in SA08, they are not for SA16 with no appreciable pattern. For both jars slight abrasive wear resulted in few scratches visible on the exterior. The morphology, performance matrix and evidence of use of jars SA08 and SA16 primarily point to functions related to **heating**,** processing and storing of liquid substances**, likely involved in fermentation mechanisms (Rice 1987; Arthur 2003; Skibo 2013; Craig 2021).

#### Other shapes

**Fig. 9:**
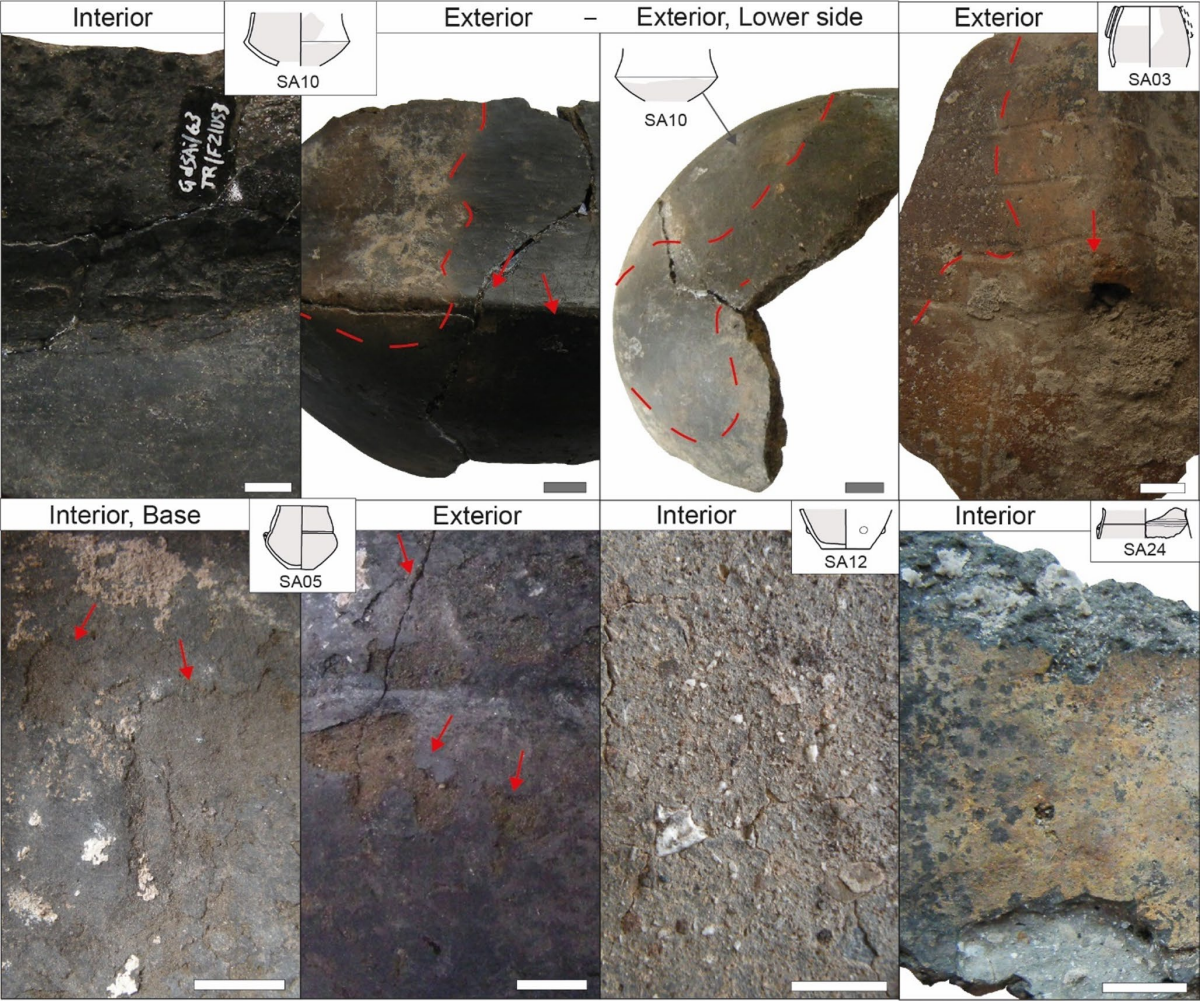
Exemplary use-alteration traces documented on various forms from Trivio area. The area interested by extensive traces is marked in gray in the drawing of the pot. Squared area devoid of material in SA24 resulted from sampling operations for residue analysis. The length of the white/gray bar is always 1 cm

The **cup/mug** SA10 (Fig. 4; Table 2) had high or medium stability depending on whether the original base was flat or rounded. The carination may have been intended to lower the center of gravity in the case of a tall vessel or a rounded base. Accessibility of its content could be estimated as medium due to the presence of carination whereas transportability is high, even for liquids, due to its small size and light weight. The pot can be held in the hands but when filled with hot content holding it could have been hampered by thermal hazards if no handle was present. No inference is possible on the containment security, since the rim area is not preserved, but impermeability can be estimated at a low level of development as the internal surface is smoothed. Regarding evidence of use, one can observe oxidation in just one area of the external surface on the upper part of the pot and discolorations largely in the lower exterior side of the vessel, towards the base (Fig. 9). The exterior further shows spall detachments and fractures, whereas the interior displays spall detachments, depressions and pits, leading to areas of pedestalling inclusions. The bowl/mug SA10 was used for **meal preparation** involving **heating** as well as **consumption of liquid food or beverages**. Consumed substances were corrosive agents affecting the pot’s internal surface.

The **pyxis** SA03 (Fig. 4; Table 2) was likely not particularly stable given the position of the suspension feature on the upper part of the restricted opening, only partially counterbalanced by the low center of gravity provided by the position of the maximum expansion. The morphology of the (currently missing) base could have enhanced or hindered the degree of stability. However, this shape has the advantage of securing the content very well, even in the case of liquids and during transportation, as the pot can be closed with a lid, cloth or skin cover, all of which could be tied to the suspension feature(s). Therefore, the accessibility of its content is low, and lip orientation is incompatible with pouring. Transportability is highly developed thanks to light weight and low capacity and the pyxis can clearly be carried either by hand or with cords, managing to transport solid, liquid, and/or hot contents. Once the contents are secured by closure, transportation could be carried out over medium to long distances too. However, it should be noted that the surfaces were regularized only by the potter and thus impermeability is poorly developed. The overall shape, with its smooth contours and relatively thin walls, enhances thermal conductivity. The external surface, including the area of the suspension feature, is oxidized and spall detachments are visible near the suspension hole and in the lower part of the pot (Fig. 9). The internal surface exhibits a faintly visible lighter dyschromia indicating the ancient level of the content. The pyxis was clearly **suspended over a fire** and used for **wet cooking**, and thus was involved in **food processing** and **meal preparation** operations.

The **beaker** SA05 (Fig. 4; Table 2) is characterized by high stability and transportability, but medium accessibility and containment security. The vessel can be carried with even one hand and can easily transport liquids with exterior lip extension that favors pouring; when filled with hot content the hold may prove problematic due to thermal hazard. The beaker can be closed by means of a lid or be covered with cloth/skin material that can be secured under the rim using a cord and additionally to the suspension feature or the horizontal groove running at approximately the midsection of the vessel. It is possible to introduce a tool in the pot or to access the content via the mouth, but the use of fingers/hands by adults is rather difficult if not impracticable. Impermeability is poorly developed due to its smoothed internal surface. The surfaces of the beaker are exfoliated by several thin spall detachments and show extensive and frequent depressions and pits, evidence of corrosive wear (Fig. 9). The beaker SA05 was likely used for **consuming liquid food or beverages**, which severely damaged the pot’s interior.

The **necked vase** SA04 (Fig. 4; Table 2) has highly reduced accessibility of the content, moderate size and weight, and can be carried with the hands or between the arms and secured against the chest; when filled with content, most likely liquid, transportation may be difficult on account of total weight. Permeability is reduced by a clay coating on the external surface which may have helped in keeping liquid content inside. Moreover, the thick walls of SA04 may have retarded the penetration of liquids into the ceramic matrix and the following evaporation. Concerning **use-related alterations**, they mark the occurrence of primarily corrosive wear with depressions and pits on the internal surface and incipient fractures likely related to fatigue wear on the interior, especially around big inclusions exposed to the surface, and on the exterior (Table 2; Fig. 8). The neck of SA04 bears testimony to the original level of the content thanks to an evident blackish to grayish dyschromia visible on the internal surface which originated from the penetration of the organic matter in the pot’s wall and its decomposition. The blackish area starts in the mid neck and extends to the vessel’s shoulder. This evidence allows to infer that the necked vase SA04 was likely used for **storing liquid content**, possibly fermented (Arthur 2003; Skibo 2013; Forte et al. 2018).

Regarding the potsherd for which the shape is **undetermined**, only limited inferences can be drawn based on **morpho-functional characteristics** (Fig, 4; Table 3). Stability is determinable for SA24 only, which has a low center of gravity ensured by low shoulder and suspension feature in a similar manner to the bowl SA14. Accessibility cannot be estimated for most of the sherds except for SA12 that shows a tendency for a medium opening allowing at least a tool to enter, and SA24 most likely had medium accessibility. Transportability is also difficult to determine and can be hypothesized as medium for SA02 and SA12 and high for SA24. The latter is also the only one allowing for determination of containment systems: the restricted shape keeps the content inside, especially liquids, and retards the evaporation during heating. A lid or stopper could possibly have been used for SA24, but suspension features are possibly placed too low to facilitate the closure with a cloth or skin cover. Impermeability is low in all potsherds as they have unfinished (SA12), regularized (SA02, SA09, SA19), and slightly smoothed (SA24) internal surfaces. **Use-alteration traces** provide useful data on the actual use of pottery (Table 3; Fig. 9). Evidence of corrosive and fatigue wear occurs on SA02, SA09, SA12 whose surfaces show fractures, depressions, and pits, with external surfaces having few spall detachments including on the area of the buttons and eventually the handle. The internal surface of SA12 is additionally featured by extensive areas of pedestalling inclusions due to corrosive wear (Fig. 9) and exhibits a lighter dyschromia in the lower interior at the base and lower part of the vessel related to the original level of content. The external surface of SA02 also has few scratches. Unfortunately, a hypothesis on the function can be proposed only for **pot SA12**, which possibly served in **processing of food**, including corrosive substances, and in **fermentation mechanisms along with storing**. The pattern of use-alteration traces for SA19 and SA24 is different, mainly showing evidence of thermal stress, such as discoloration of external surfaces and spall detachments on the interior. SA24 further exhibits fractures, cracks, spall detachments, and pits on the exterior, as well as spall additional detachments, fractures, and a patina on the interior (Fig. 9). **Pots SA19** and **SA24** were possibly used for **cooking** and were involved in **food processing** and **meal preparation**.

### Lipid extracts

**Fig. 10:**
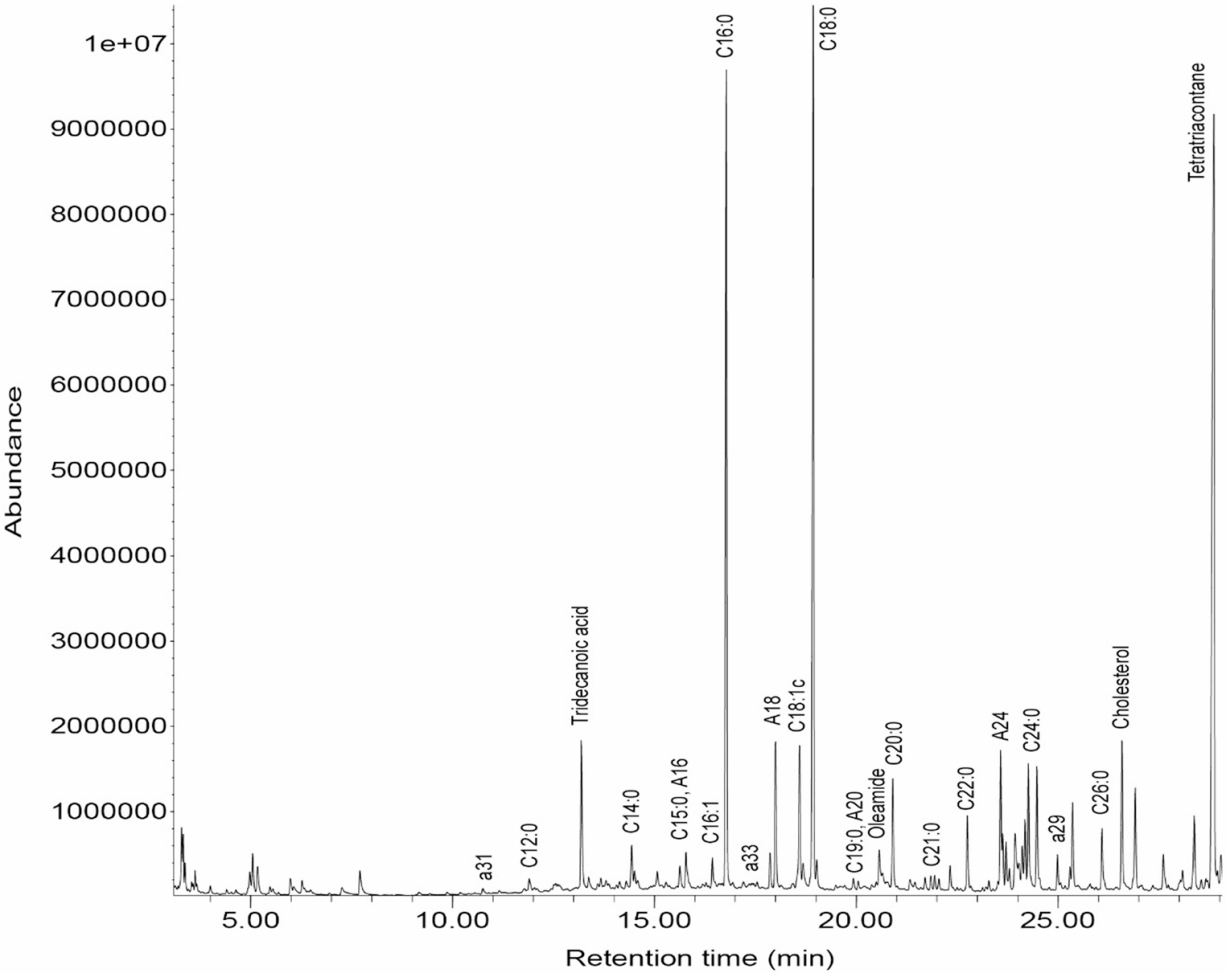
Exemplary total ion chromatogram obtained from the restricted bowl SA14, interior layer S2; tridecanoic acid and tetratriacontane were introduced during sample preparation as internal standards

All the analyzed sediments and pots from the Trivio area yielded lipid residues of different classes and measured in variable amounts. Figure 10 shows an exemplary GC-MS chromatogram, whereas Supplementary Information 2 contains detailed data on sediments and pots’ lipid profiles (both S1 and S2). With reference to pottery, no significant difference exists between S1 and S2 samples nor in terms of presence/absence or of peak intensity of specific molecules. The only variation concerns the measured total lipid concentration in µg/g which is regularly higher in S1 samples as archaeological residues are mostly located within a depth of 2 mm of the ceramic interior (Stern et al. 2000; Steele and Stern 2017). No evidence for the use of adhesives to enhance impermeability has been found; therefore no differentiation is applied between S1 and S2 samples of the same pot. This section provides a general overview of lipid classes documented in pottery and sediments and their significance as archaeological biomarkers.

Pottery samples yielded TLE values ranging from approximately 24 to 442 µg/g, whereas sediments showed slightly lower concentrations, between approximately 30 and 195 µg/g (Supplementary Information 2). Pots exhibit higher lipid concentrations than the sediments collected at their findspot. Lipid profiles report the following relevant lipid classes: fatty acids, fatty alcohols, alkanes, terpenoids, sesquiterpenoids, wax esters, steroids, and fatty amides (Supplementary Information 2). Lipid profiles of pots are dominated by fatty acids, showing higher peak intensities compared to the other molecules registered in the chromatograms (e.g. Figure 10); exceptions to this general trend are SA06 and SA09 where peak areas of fatty alcohols are higher than the ones of fatty acids and SA08 and SA13 where, along with fatty acids, fatty alcohols and fatty amides are important as well. Other lipid classes are minor and can be detected only by small peaks. In the lipid profiles of sediments, fatty acids tend to not dominate, and proportions of lipid classes vary across the sample set (Supplementary Information 2). Fatty alcohols prevail in chromatograms of F2 US2, F2 US3, and P US1, and their proportions are like those of fatty acids in US1 deposit from findspots 3 and 14. The sesquiterpenoid longiborneol is remarkably present in US 1 sediments from F2, F3, F4, and P21. Conversely, this molecular species is basically absent in the set of the absorbed residues by pots, being represented in chromatograms only by very minor peaks. Among the non-lipid constituents, phosphate is regularly present in sediment samples, and even dominates the range of detected molecules in the case of findspot 9 US1 but is less incisive in pottery profiles. Finally, fatty amides are relevant component of the US 1 sediments of findspots 14 and F3 and are present in all chromatograms from pots except SA01 and SA02, with particularly high peak intensities in pots SA04, SA08, SA13, and SA16.

The kinds of residues detected in sediments and pots (Supplementary Information 2) represent valuable **archaeological biomarkers** of natural resources. Short to very long chain saturated fatty acids can be generally related with animal resources such as adipose or milk/dairy products (Evershed et al. 2002; Regert 2011), whereas linear and branched odd-numbered saturated fatty acids originated specifically from ruminant animals, being markers of the bacterial population living in the rumen (Evershed et al. 2002). The presence of cholesterol is possibly related to degraded animal fats although this steroid is highly prone to oxidation and tends to not preserve its original structure over archaeological timescales (Whelton et al. 2021). Unfortunately, it is not possible to exclude the contamination from skin lipids due to recent human handling of the sampled material as the origin of cholesterol residues. This is because the characteristic degradation products of original cholesterol are not clearly visible in the analysed samples, while traces of squalene were detected (Reber 2022). Pristanic and phytanic acids are diterperoids indicative of aquatic resources, including fish (Hansel et al. 2004; Regert 2011; Lucquin et al. 2016). Vegetal resources may be inferred from presence of unsaturated fatty acids – mainly C18:1c – steroids such as sitosterol, campesterol, and stigmasterol, alkylresorcinols, and terpenoids (Heron and Evershed 1993; Evershed 1993; Cramp and Evershed 2015; Reber 2022). In particular, alkylresorcinols are category biomarkers of cereals and terpenoids include both resource-specific compounds, such as betulin from birchwood and longiborneol from coniferous, and general category biomarkers (Charrié-Duhaut et al. 2007; Colonese et al. 2017; Sarret et al. 2017; Hammann and Cramp 2018; Rageot et al. 2021). Dehydroabietic acid may have originated from *Pinaceae sp.* resin, but the absence of co-occurring pimaric acid hinders its reliability as a valuable archaeological biomarker (Steele and Stern 2017). Wax molecules detected in pots SA09, SA12, SA14, and SA22 may refer to both vegetal and animal resources with plant epicuticular waxes traced back thanks to waxes W38–W40 and their alteration products, such as fatty alcohols and alkanes, resulting from hydrolysis (Evershed et al. 1991; Evershed 2008; Reber 2022; Drieu et al. 2022). With reference to beeswax, evidence is not straightforward being limited to occurrence of waxes W42–W48 in pots SA09 and SA22 (Heron et al. 1994; Evershed et al. 2003). Fungal substances are represented by ergosterol (Isaksson et al. 2010), detected only in some US1 sediment samples; these may be related to the overall cave ecosystem or its contamination by recent human presence (Poli et al. 2024), rather than being part of the archaeological findings. Presence of hopanoids such as neogammacerane, neonorgammacerane, and hopane occurring in most, but not all, sediment and pottery samples in traces are more difficult to frame in the current state of knowledge, often reported as products of fermentation or biomarkers of beer, whereas bitumen use can be excluded due to the absence of steranes and aromatic hydrocarbons (Jones et al. 1999; Connan et al. 2004; Guerra-Doce 2015; Faraco et al. 2016; Roffet-Salque et al. 2017; Nardella et al. 2019; Pennetta et al. 2020a). Fatty amides are compounds both naturally occurring and plastic-derived (Reber 2022). These were detected in some sediments and pottery from the Trivio area in the form of stereamide, oleamide, and palmitamide (Supplementary Information 2), with oleamide consistently being quantitatively predominant over the others. The fatty amides can be produced by heating fatty acids with amines to a temperature of 200 °C and thus can be an indicator of food cooking (Anta and Kennedy 1991). However, oleamide is also a lubricant and may have absorbed by samples due to conservation in plastic bags in the archaeological deposit (Whelton et al. 2021). Lastly, phosphate could have been absorbed by pots in the burial context or can be considered a nonspecific indication of use of the pots, being a possible residue originated from plants and animals (Duma 1972; Dunnell and Hunt 1990; Heron and Evershed 1993).

The targeted analysis of **fermentation products** provided information on the presence of carboxylic acids and phenolic compounds in a subset of samples: SA01, SA03, SA04, SA05, SA08, SA10, SA12, SA16, SA25 (Supplementary Information 2). Succinic and malic acids were detected in all samples in the range of 0.6–4.64 µg/g and 0.5–2.35 µg/g, respectively, with SA04 showing the higher concentration of the former. Additionally, SA12 showed the higher amount of malic acid. Lastly, tartaric acids and syringic acids are present only in traces (< 0.24 µg/g), with tartaric–malic ratio that never exceeds the value of 0.17. Considering obtained data, this additional test indicates that the analyzed pots contained fruit-related products with no specification of the species as carboxylic acids and phenolic compounds are category, not resource-specific, biomarkers (Cramp and Evershed 2015; Whelton et al. 2021; Reber 2022). Tartaric acid, typically considered an indicator of grape or wine, is present only in small concentrations and with a non-diagnostic tartaric–malic ratio; therefore, it should be regarded as a general marker of fruit consumption (Michel et al. 1993; Pecci et al. 2013; McGovern et al. 2017; Perruchini et al. 2018; Drieu et al. 2020; Whelton et al. 2021).

**Fig. 11:**
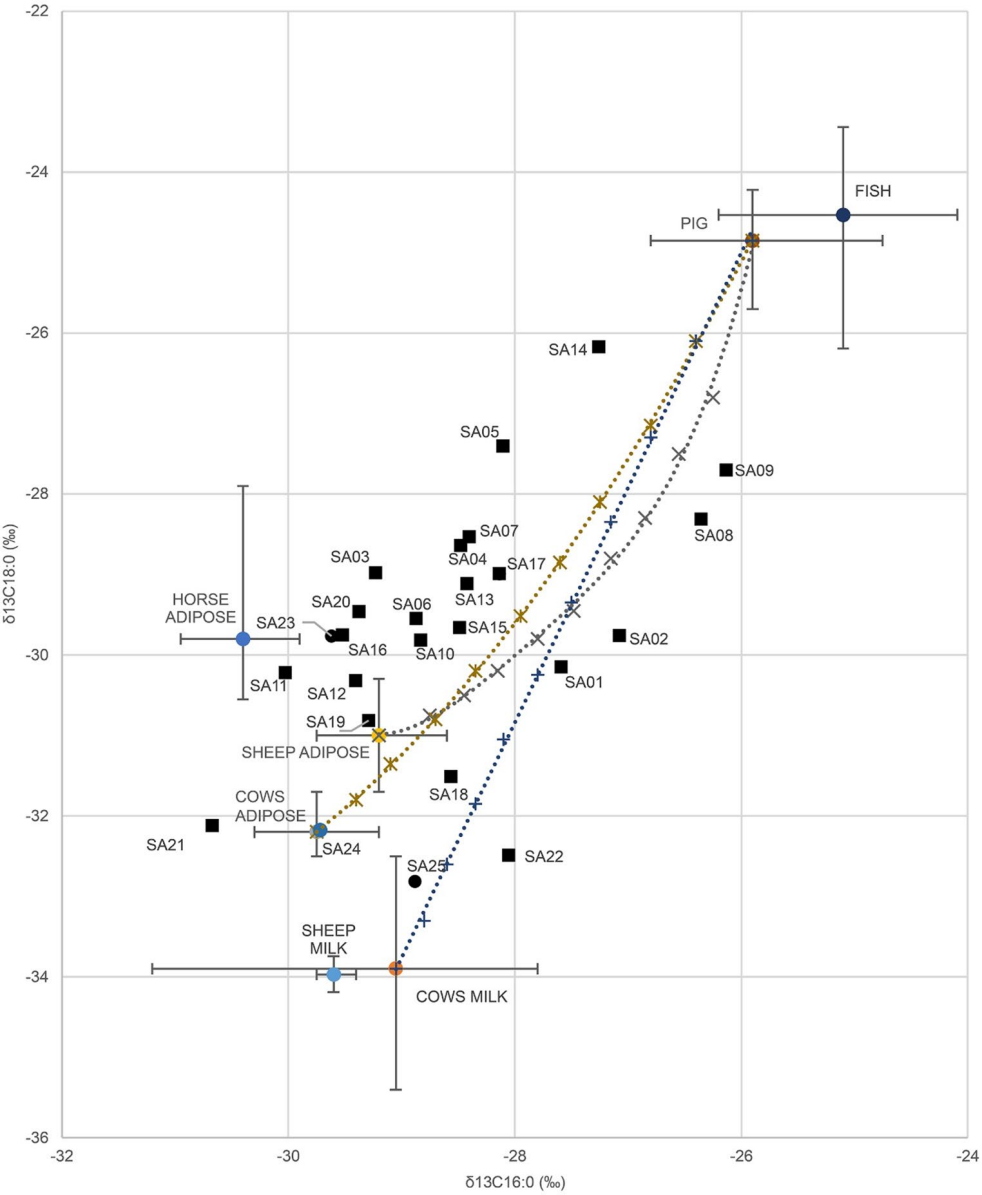
Plot of the δ^13^C values of C16:0 and C18:0 fatty acids preserved in pottery from Trivio area and reference values for animal products from Copley et al. (Copley et al. 2003), Craig et al. (Craig et al. 2012), Evershed et al. (2016). The mixing curves illustrate calculated δ13C values for the mixing of ovine and porcine fats (x), bovine and porcine fats (✶) and cow’s milk and porcine fats (+) in the vessels

GC-C-IRMS provided information on the **stable carbon isotope** ratios of methyl-palmitate and methyl-stearate and enabled differentiation of animal fats. Obtained δ^13^C values of the C16:0 and C18:0 fatty acids from pottery samples are reported in Supplementary Information 2, whereas Fig. 11 shows data from Trivio pottery in the framework of reference δ^13^C values for animal fat of Mediterranean prehistoric interest (Evershed et al. 2002, 2016; Copley et al. 2003; Craig et al. 2012; Budja 2014). Isotopic analysis of pottery yielded δ^13^C16:0 ranging from – 30.67‰ to – 26.13‰ and δ^13^C18:0 between − 32.81‰ and − 26.17‰, with variable Δ^13^C (corresponding to δ^13^C18:0–δ^13^C16:0). The obtained values suggest a predominance of mixtures of fats in the dataset (Fig. 11). More precisely, most samples cluster along around the mixing curves between ruminant and non-ruminant adipose, following two distinct patterns: (1) ovine and porcine fats for SA02, SA08, SA09 and (2) bovine and porcine fats for SA04, SA06, SA07, SA10, SA13, SA15, and SA17. Pots SA01, SA18 and SA22 show Δ^13^C values compatible with the presence in the mixture of dairy products. Only a few pots display an isotopic signal overlapping to a single source type, i.e. SA25 with dairy products, SA12 and SA19 with sheep adipose, and SA21 and SA24 with cow adipose. Apparently, pot SA11 yielded a Δ^13^C value matching that of the reference horse fat, but it could also contain a mixture of lipids from different sources, whose average isotopic values produce a composition relatively close to equine adipose tissue. Finally, six other measurements of archaeological fats plot along areas of mixed values for which reference data are currently lacking (SA03, SA05, SA14, SA16, SA20, SA23, see Fig. 11).

**Fig. 12:**
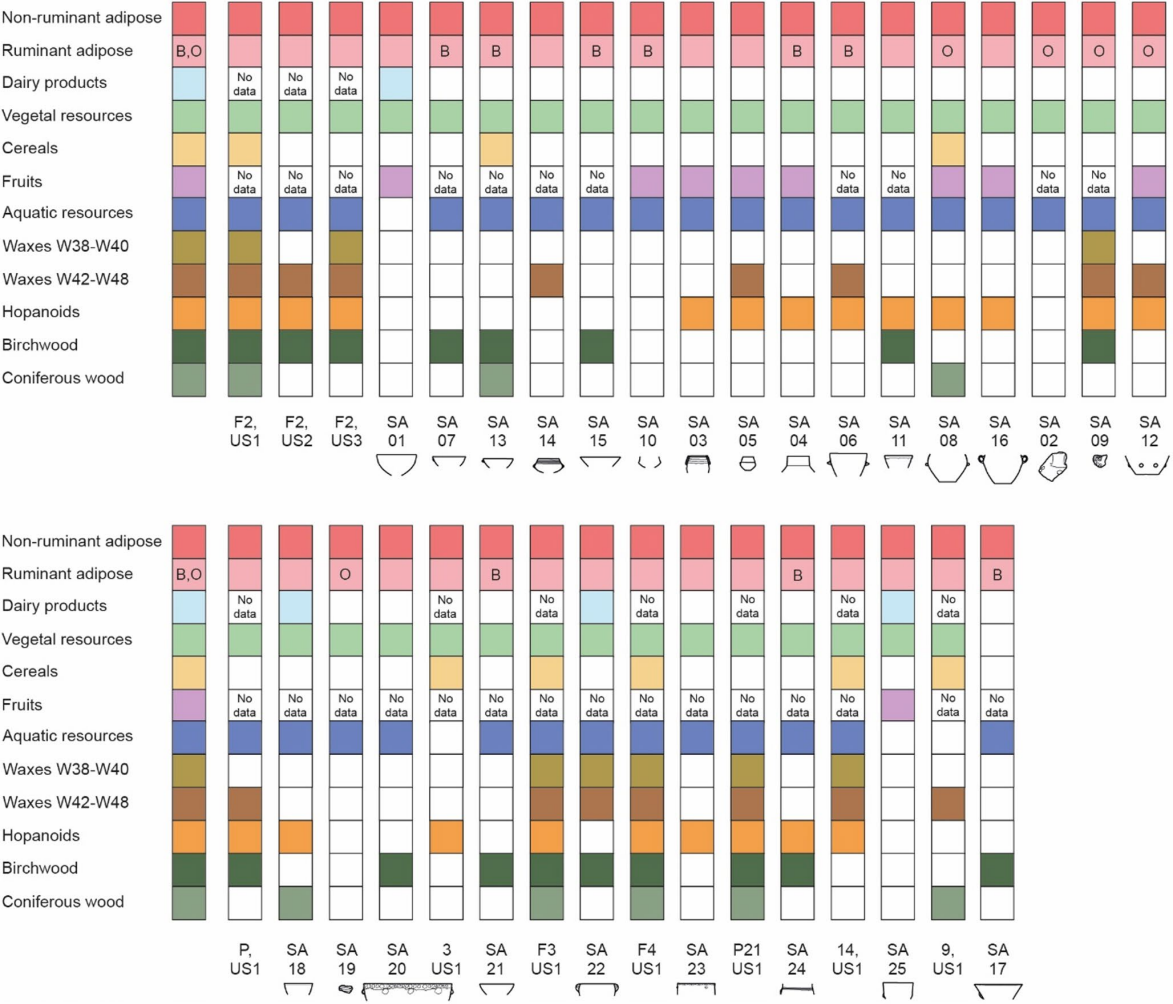
Types of lipid biomarkers detected in sediments and pottery from the Trivio area with empty squares indicating absence of specific resources through the sample set. Ruminant adipose is detailed as from bovine (B) or ovine (O) species when possible, whereas squares marked as “No data” clarify that the sample was not tested for the presence of that particular resource

In conclusion, Fig. 12 summarizes the interpretation of residues found in sediments and pottery from the Trivio area. To facilitate the following discussion of results from analysis of lipid extracts the list is arranged by pottery’s findspot in the Trivio area (Fig. 2; Table 1) and shape (Fig. 4). By comparing GC-MS and GC-C-IRMS data it emerges that in samples SA22 and SA25 – whose isotopic signal indicates dairy products – the presence of non-ruminant adipose cannot be excluded, though it was likely not significant. Similarly, samples SA03, SA05, and SA15 show traces of ruminant fats, evidenced by the presence of linear and branched odd-numbered saturated fatty acids; however, their stable carbon isotope ratios indicate that they mainly contained non-ruminant fats.

## Discussion

The chrono-cultural and functional study of ceramic containers from the Trivio area of the Grotte di Sant’Angelo Cave Complex made it possible to detail the kinds of pots brought to the cave and their significance in relation to material culture, technological know-how, and the exploitation of edible natural resources (Sect. Results). In this section, we use the information acquired on pottery artefacts from the case study to explore the six contextual dimensions of cave relevance identified by Bergsvik and Skeates (2012a) and to evaluate the potential of using in-depth pottery studies for the development of European cave archaeology. Table 4 summarizes the most important contextual findings of the case study and highlights the potential of the proposed techniques for advancing European cave archaeology. Below we address the six dimensions.

**Table 4:** Summary of the contribution of the chrono-cultural and functional study to the contextual interpretation of the case study according to Bergsvik and Skeates (2012a, b) and evaluation of the potential of the proposed techniques in the development of European cave archaeology.

	**CONTRIBUTION OF POTTERY CHRONO-CULTURAL AND FUNCTIONAL ANALYSIS**	
**CONTEXTUAL DIMENSIONS**	**CASE STUDY**	**CAVE ARCHAEOLOGY**
**Stratigraphy and preservation** *Values of caves for stratigraphic resolution and sometimes favourable conditions for preservation of organic material*.	The Trivio area does not represent a good stratigraphic context, but a good preservation one for ceramic morphology, surfaces and organic residues. The prehistoric pots abandoned in the cave likely contaminated the sediments within the depositional context. Other lipid categories provided evidence of additional human activities in the cave, not necessarily occurred in prehistoric times.	Assessment of the value of the cave in terms of stratigraphy formation and preservation of original artefacts’ morphology, surfaces and of organic matter associated with them. Evaluation of the effects of anthropization of cave spaces on the underground ecosystem and on the archaeological depositional context throughout time.
**Temporal context** *History of cave occupation, transformation, and remembrance (or forgetting) both seasonally and over the long-term of centuries and millennia*.	Prehistoric human presence in the Trivio area dates to the entire 4^th^ millennium BCE, possibly without solutions of continuity. The pots brought to the cave were used for cooking, warming up/keeping food warm, serving/processing/consuming, storing, and transporting/transferring, with no evidence of distinction based on the phase of the Copper Age period. First outline of the history of occupation.	Definition of the timeframe of human presence in the cave, articulation in phases and eventual solutions of continuity. Identification of the pots brought to the cave by period and of their past content as part of the general inquiry into the activities conducted at the site. Outline of the history of occupation.
**Architectural context** *Natural and cultural formation processes (or ‘speleogenesis’), and their typological relation to other architectural forms (such as megalithic tombs), framing, affecting and adding significance to human activities*.	Ceramic containers found in the cave tended to be easily to moderately transportable and could be carried in a variety of ways. However, moving around this sulfuric cave space while holding pots was probably not easy and risky. Pottery vessels are all related activities that cannot be comfortably or safely performed in the Trivio area and at their findspots. The sulfuric cave physicality somehow impacted on people’s physical, sensory, and spiritual experiences and perceptions. Ceramic containers and preserved organic residues, along with fauna remains, may suggest consumption of food in the cave before abandonment in the cavities on the floor. However, evidence in this regard is not conclusive, and data from additional research areas are needed.	Discussion on how the ceramic containers were brought to caves considering underground ‘architecture’, possible paths, degree of ease of movement through cave spaces, morpho-functional properties of ceramic containers and their past content. Reflections on the suitability of cave spaces for performing the activities evidenced by use-alteration traces on the pots and insights on human-cave entanglements and role played by non-human agencies in these (e.g. the speleogenetic processes). Speculation on the possible activities conducted in the cave and their association with spheres where abstract thinking plays a major or minor role.
**Spatial context** *Relevance of caves as architectural spaces and as meaningful places in the landscape, connected to (or maginalized from) other landforms, resources, and patterns of human behavior*.	The pottery from the Trivio corresponds to assemblages from Copper Age sites located in Southern and Central Italy and acquired data reinforce the idea that caves may have been particularly important for Copper Age societies. The types of food resources introduced in the cave were procured and probably processed in the aboveground realm.	Place of the cave in the larger context of known sites, patterns of human occupation of landscapes, circulation of cultural models, and material culture distribution. Definition of the types of food resources introduced in the cave and their origin from the underground/aboveground realm.
**Socio-economic context** *Meaningful place of caves within wider cosmologies, ritual actions, economic strategies, social practices, power relations, identities, and memories*.	Certain stylistic aspects of ceramics – traditionally holding a high chrono-cultural value for Southern Italy – have also a functional meaning. Copper Age people frequenting the cave used to cook food resources by both suspending the pot over the fire and not, in both wet and dry modes. Fermentation is an additional technique of food processing. Residues point out 4^th^ millennium BCE people consumed food resources of different kinds: animal adipose from both ruminant and non-ruminant species, aquatic, vegetal resources, dairy products, birchwood, and coniferouswood. Dairy products were found in deep, unrestricted bowls. Adipose from equids is suspected, but currently not confirmed. The pots were surely used, probably multiple times, before being abandoned in the cave, pointing out that the ceramic containers were not specifically fabricated for abandonment in the cave. Contextualization of evidence from the Grotte di Sant’Angelo Complex within the known ritual actions performed by Copper Age people in Southern and Central Italy.	Relation between stylistic and functional aspects contributing to the reconstruction of past identities. Insights into ways of processing food resources for nutrition purposes as part of the general economic strategies. Revelation of the kinds of food resources procured and consumed by past population adding to the knowledge on general economic strategies. Evaluation of the degree of use-related alterations to determine whether the pots were specifically fabricated for abandonment in the cave or were first extensively used. Contextualization of acquired data from pottery within the wider framework of known ritual actions conceived and performed by past population in relation to both underground and aboveground realms.
**Scholarly context** *Place of cave in the dynamic history of science and of archaeology*.	Acquired data on pottery from Trivio challenge the traditional assessments of the archaeological value of the Grotte di Sant’Angelo Cave Complex, which were based on the culture-historical approach.	Identification of weaknesses of traditional/previous interpretations of human-cave interactions and suggestion of new research angles to test.

### Stratigraphy and preservation

Given the very low sediment production rate at the Grotte di Sant’Angelo Cave Complex – typical of the sulfuric acid speleogenetic process – the stratigraphic dimension could not be explored. Conversely, the cave offered optimal preservation conditions for archaeological remains and organic residues. As demonstrated in the study, the preservation of pottery remains allowed us to recognize features diagnostic of chronology, observe use-alteration traces, and identify a range of residues absorbed during pottery use, which serve as biomarkers of ancient foodways. Lipids were detected also in the chromatograms of samples collected from archaeological findspots, but their presence in the cave sediments shows that the pots contaminated the depositional context rather than the reverse. This is primarily evidenced by the fact that lipid concentrations are higher in pottery than in sediments at the findspots (Supplementary Information 2). Hence, the past contents of the pots, along with faunal remains and other tools found at the site, likely contaminated the cave sediments through human activities or deposition after abandonment. This inference is further supported by the presence of lipid classes in the sediments that are incompatible with the cave ecosystem, particularly in the dark zone. These include, for instance, branched odd-numbered saturated fatty acids from ruminants, pristanic acid from aquatic resources, and various plant-related lipid biomarkers. Interestingly, lipids from US 1 show a recurrent abundance of longiborneol, derived from coniferous wood, at major openings on the cave floor (F2, F3, and F4) and at findspots P21 and 9 (Fig. 2). This may be related to the use of torches or the placement of wooden walkways, which may not necessarily date to prehistoric times. The application of residue characterization work to pottery remains, combined with comparisons to sediment lipid profiles, thus provides valuable insights into the anthropization of cave spaces and the inner cave dark zone.

### Temporal context

The chrono-cultural analysis effectively defined the timeframe of human presence in the Trivio area during Prehistory, and the acquired data enable us to take a first step toward investigating the temporal context of the Grotte di Sant’Angelo Cave Complex. Thanks to this study, we can now assume that the Trivio was frequented during the 4th millennium BCE, corresponding to the Early–Middle Copper Age in Southern Italy. Combined with the functional study, these results allow us to identify the types of pots brought to the cave and begin to outline a history of occupation in the Trivio area, at least during Prehistoric times. This research revealed the earliest human presence in the Grotta di Sant’Angelo I sector, dating to the beginning of the 4th millennium BCE. This is evidenced by the beaker SA05, suitable for consuming liquid food, which was abandoned in the long fracture F2 of the Trivio area. Frequentation of this cave zone continued and intensified in the Early Copper Age and extended to the Middle Copper Age, with the introduction of pots for cooking, warming up/keeping food warm, serving, processing, consuming, storing, and transporting/transferring. The vessels were deposited in various cavities on the cave floor though especially inside the major fracture F2. No functional distinction between pottery types across the phases of the Copper Age emerged from this study.

### Architectural context

The kinds of pots found in the Trivio area contribute to understanding how the specific morphology of the Grotte di Sant’Angelo Cave Complex affected Copper Age people and how they, in turn, interacted with it. The ‘architectural’ context was introduced in Sect. Archaeological background, where the speleogenetic processes that led to the formation of the cave spaces and their general morphology were described. Contextualizing the pottery data and discussing them in relation to the physicality of the cave and its overall vibrancy *sensu* Prijatelj and Skeates (2019) provides further insights. These concern the transportation of pots inside the cave and the compatibility between the activities inferred from use-alteration traces and the cave environment. As such, they delineate future areas of investigation.

The pottery analysis revealed that, with the exception of jars SA08 and SA16, all ceramic containers had high to medium transportability and could be carried by hand, between the arms, or with cords. Nevertheless, moving through the cave while holding pots was probably not easy. Prehistoric people likely accessed the level of the Grotte di Sant’Angelo Cave Complex where the Trivio area is located through a descending rock opening, now partly obliterated by sediments (Figs. 2a and 3f). From this original entrance, one entered almost immediately into complete darkness and could take two diverging directions (Fig. 2a). To the right, there is a narrow rectilinear passage, whose floor contains an opening running along its main axis, the relic of a feeder/crevasse. To the left one could advance along a wider, easier-to-walk passage leading to the Trivio area, which is marked by the so-called F2 longitudinal fracture. Excavations in the deposit of F2 yielded abundant archaeological material. Longitudinal fractures on the floor – very frequent in the Grotte di Sant’Angelo Cave Complex (Fig. 2a) – make movement dangerous, as they increase the risk of slipping or leg injuries. The Trivio area is in fact the first larger room encountered along the path described, but it is neither comfortable nor safe. The cave bedrock floor is highly irregular, with depressions and holes that make progress difficult and hazardous. It is therefore unlikely that this space was used for ordinary, secular daily life activities, related to subsistence. The underground environment lacks not only natural light but also safe areas for sitting or sufficient space for frequent fires.

Several pots from the Trivio area exhibited thermal alterations resulting from proximity to fire or suspension above it during use. At present, it is not possible to determine whether these containers were heated inside the cave; however, future research may investigate the presence of fireplaces in the Trivio area. For now, it is important to note that prolonged wood burning in this zone would have significantly reduced the breathable air. Thus, cooking operations could have occurred outside the cave, in the aboveground realm. In any case, the archaeological artefacts found in the sector I of the Grotte di Sant’Angelo Cave Complex in the openings on the cave floor – pottery, faunal, querns, and lithic industry – are all associated with food procurement, processing, and consumption. These are traditionally classified as ‘domestic’ activities requiring light and a suitable floor morphology to be carried out comfortably. Their discovery in an inhospitable and unsafe dark cave zone, along with their abandonment in holes and openings in the rocky floor, is difficult to interpret without considering the vibrancy of the underground spaces (Clottes 2012; Dowd 2015; Dowd and Hensey 2016; Prijatelj and Skeates 2019). The sulfuric acid speleogenetic process, with its crevasses on the floor, acted as a non-human agency and may have fascinated prehistoric people even more than the underground context itself. This may explain why artefacts were deposited in holes and fractures. The floor openings of the Grotte di Sant’Angelo Cave Complex still represent spaces of indefinite termination – what Hein Bjerck called “realms beyond the reach of humans” (Bjerck 2012, p. 48): points beyond which human bodies mostly cannot pass, but where smaller animals can enter and natural powers can traverse and which exerts power on human mind (Prijatelj and Skeates 2019). These aspects of human engagement relate closely to the subjective and social experience of cave spaces and depend on abstract thinking in the cultic and ritual spheres. Finally, the ceramic containers, with their preserved organic residues, together with faunal remains, may indicate food consumption in the cave before the artefacts were abandoned in the floor cavities. This scenario is not unlikely for Copper Age people (e.g. Milisauskas and Kruk 2011a, b). However, it is not possible to state what was surely brought and eventually consumed in the cave based on residue characterization as lipids accumulate throughout time during use. As shown by our traceological study, the pots were very likely reused multiple times (Skibo 2013; Reber 2022). Additional data – particularly from future archaeozoological and archaeobotanical research – will be necessary to refine this interpretation.

### Spatial context

With regard to the spatial context of the Grotte di Sant’Angelo Cave Complex, the results of this study contribute to addressing two aspects: (1) the spatial distribution of 4th millennium BCE archaeological sites and material cultures, and (2) the origin of documented food resources in the aboveground, open-air realm.

Concerning the first issue, the chrono-cultural study of pottery from the Trivio area revealed contemporaneity with multiple sites in Calabria, both in caves and in open-air settings, thereby adding to our understanding of patterns of human habitation (Fig. 6). In Northern Calabria, it is particularly important to note the correspondence with other underground contexts on the Monte San Marco hill – Grotta di Sant’Angelo III, Grotta Pavolella – and with the following caves: Grotta di San Michele a Saracena, Grotta del Caprio, Grotta della Madonna, Grotta Cardini, Grotta della Monaca, Grotta del Tesauro, and Grotta della Camastra (Bernabò Brea et al. 1989; Bernabò Brea and Cavalier 2000; Tiné and Natali 2004; Larocca 2005; Breglia and Arena 2015; Ippolito 2016; Natali et al. 2021). These contexts attest to human presence in the underground realm for domestic, cultic, and mining purposes. As for open-air sites, only a few dating to the Copper Age periods are known in Northern Calabria (Tiné 1987; Pessina and Tiné 2008; Pacciarelli 2011; Breglia and Veneziano 2021). This predominance of 4th millennium BCE cave sites over open-air ones persists even though certain areas have been intensively surveyed over the years (van Leusen and Attema 2002; Attema and van Leusen 2004; Attema et al. 2012; Attema and Ippolito 2017; Ippolito and Attema 2018; De Neef et al. 2021). In this regard, the evidence from the Grotte di Sant’Angelo Cave Complex reinforces the idea that caves may have been particularly important for Copper Age people (Pacciarelli 2011; Breglia and Veneziano 2021). Among the known open-air sites (Ippolito 2016), sufficient data exist to state that the pottery from the Trivio area is contemporaneous with Copper Age occupation at Serra Cagliano 1 (Guerzoni and Amodio 2011) and, to some extent, with Middle Copper Age phase at Acri (Castagna and Schiappelli 2004). Moving further south into Central Calabria, the number of known open-air sites is much higher and predominant (Pacciarelli 2011; Breglia and Veneziano 2021). As demonstrated in Sect. Chronology of pots, the Copper Age pottery from the Trivio area corresponds to ceramic assemblages from the Tropea Promontory and the Sila Massif based on common decorative styles and the presence of subcutaneous suspension features. These typological characteristics were widespread over a wide geographical area during the 4th millennium BCE and attest to a broad circulation of cultural models affecting communities in Sicily, Calabria, Puglia, and Basilicata, extending to Campania and even to the area of Rome in Central Italy. It should be noted that, despite these important similarities in pottery typology, differences among the various sites remain evident. This supports previous observations by Pacciarelli (2011) and Pacciarelli and Talamo (2011) that, in Southern Italy, the cultural relationships and chronological connections between the different phases of the Copper Age are still only roughly understood. Based on the results of the chrono-cultural study, it is therefore possible to contextualize the pottery from the Grotte di Sant’Angelo Cave Complex within a broader framework of material culture formation, articulation, and spatial distribution.

With regard to the second issue, the functional analysis revealed that the food residues documented in the ceramic containers from the case study are, as expected, all related to food resources procured and probably processed in the aboveground realm. Animal, plant, and aquatic resources, birchwood and coniferous wood, milk and dairy products, fruits and cereals need natural light, oxygen, and abundant water to thrive. This suggests the dependency of the cave deposit on the life and activities conducted by 4th millennium BCE communities in the open-air. Placing the Grotte di Sant’Angelo Cave Complex within the wider context of prehistoric populations and their above-ground landscape, as well as analyzing the cave’s position in relation to resource distribution, will require contributions from other fields of research, such as environmental archaeology.

### Socio-economic context

The acquired data on pottery findings further contribute to exploring the socio-economic context of the Grotte di Sant’Angelo Cave Complex. First and foremost, this study revealed that certain stylistic aspects of ceramics – traditionally considered diagnostic for chronology and cultural attribution in Southern Italy – also had a functional meaning and resulted from significant technical investment aimed at manufacturing containers capable of performing intended functions (Sect. Morpho-functional characteristics and visible use-alteration traces ). This is clearly visible, for instance, in the use of subcutaneous handles in relation to the practice of suspending pots over the fire or in the fact that Early Copper Age pots used for proper cooking were all decorated with horizontal parallel grooves (SA14, SA24 and possibly SA19). Moreover, striations left by cords around buttons and listels suggest that these important chrono-cultural markers were possibly related to the need of closing the pots with lids or cloth/skin. Similarly, the presence of perforated lugs in the jar SA06 is associated with the need to move the pot during dry cooking operations, such as toasting or roasting food resources. Other evidence adds to current knowledge of Copper Age pottery, material culture, and foodways on which the cave archaeological deposit depends. A key inference from this study concerns the ways Copper Age people used cooked food resources, both by suspending pots over the fire (restricted bowl SA14, pyxis SA03) and by placing them directly on or near the fire (jars SA06, SA20, SA23), employing both wet and dry cooking modes (Sect. Morpho-functional characteristics and visible use-alteration traces). Moreover, the functional study of the bowls and the cup/mug revealed an unexpected insight: these open shapes were not only used for serving and consuming meals, as their morphology suggests, but also for preparing them by heating. Essentially, all unrestricted bowls dating to the Early and Middle Copper Age, except for SA07 and SA15, were used for warming up the content or keeping them warm. An additional insight concerns the practice of storing, transporting, and pouring broth or stock and/or fermented beverages (necked vase SA04, jars SA08 and SA16, and base SA12). Jars SA08 and SA16 were also likely used for their preparation through mild heating. With reference to the types of food resources procured and consumed during the 4th millennium BCE, the study provided evidence of provisions from the animal, plant, and aquatic realms. Animal adipose from both ruminant and non-ruminant species, as well as aquatic and vegetal resources, recurs in the analyzed set of pots with no distinction based on shape (Fig. 12). The detected residues from cooking pots generally match those found in vessels used for meal consumption or drinking throughout the entire period. However, the situation is different for dairy products, which were definitively identified only in the lipid profiles of deep, unrestricted bowls (SA01, SA18, SA22, and SA25; Fig. 12). Consumption of milk from ruminants is not new in prehistoric Calabria region, as lipid residue analysis of 6th millennium BCE *Impressa* pottery from Grotta di San Michele di Saracena has already shown (Debono Spiteri et al. 2016). Other food resources documented in the ceramic lipid profiles are rather peculiar. Birchwood may suggest the use of birch-bark for flavoring purposes^3^ (Reber 2022), whereas the presence of coniferous wood in jar SA08, may indicate its involvement in the preparation of fermented products. Finally, Middle Copper Age jar SA11 possibly bears testimony to the consumption of adipose from equids – an inference that cannot be currently confirmed in the absence of relevant archaezoological data^4^. Unfortunately, all these considerations on foodways cannot be compared with data from open-air contexts due to the general lack of knowledge regarding aboveground sites in the region.

Another important aspect to consider is that the traceological study demonstrated that the pots were certainly used – likely multiple times – before being abandoned in the cave, as the abundance of use-alteration traces is incompatible with a single use. This indicates that the ceramic containers found in the Trivio area were not specifically fabricated for the sole purpose of being deposited in the cave. At the current state of research, it is not possible to discuss whether the pots were abandoned as complete vessels with their contents, or if they were empty or in a fragmented state. It is worth noting that no pottery shows signs of repair, reuse, or recycling and that faunal remains were found in association with archaeological objects (Sect. Archaeological background).

Still inferring on the socio-economic context of the Grotte di Sant’Angelo Cave Complex, and considering the archaeological deposit of the Trivio area as possibly resulting from activities predominantly related to the ‘sacred’ sphere, it should be noted that pots played a significant role in cultic activities in caves throughout prehistoric Central and Southern Italy, especially in relation to underground water (Whitehouse 1992; Skeates 1994; Bernabei and Grifoni Cremonesi 1995; Pacciarelli 1997; Grifoni Cremonesi 2007; Gullì 2013, 2018; Larocca and Breglia 2014; Larocca 2018; Breglia and Veneziano 2021). In addition, during the 4th millennium BCE ceramic containers also materialized rites in the open air, being deposited in excavated pits in the ground as offerings, e.g. at the sites of Piano di Cecita in the Calabria region and Casetta Mistici Osteria del Curato-via Cinquefrondi in the area of Rome (Bernabei and Grifoni Cremonesi 1995; Anzidei and Carboni 2020; Marino and Nicoletti 2023). At the current state of research, it remains unclear whether only certain types of pots were brought to caves, as more data are needed on the characteristics of pottery assemblages from settlement and funerary sites. As discussed in relation to the spatial context, the situation is even more complicated for the regional framework of the Grotte di Sant’Angelo Cave Complex, as more cave sites are known than open-air ones. Undoubtedly, while the concealment or offering of pots and other everyday objects in cave sites is a known practice in prehistoric Central and Southern Italy, the concealment of artefacts in the openings of a cave’s floor bedrock is a newly identified behaviour. This discovery, along with the data collected from the chrono-cultural and functional characterization of ceramic containers, opens new research perspectives on the engagement of prehistoric populations with the peculiar physicality of caves formed by sulfuric acid speleogenesis, as well as the meaningful place of caves within broader ritual practices.

### Scholarly context

Regarding the scholarly context, the data collected should also be discussed within the framework of the history of research on the Grotte di Sant’Angelo Cave Complex. Their archaeological relevance was first explored in the 1960 s, through the excavations conducted in sector Grotta di Sant’Angelo III, during a period when the culture-historical approach dominated research. Consequently, the excavator focused primarily on stratigraphy and on the necessary definition of archaeological cultures based on artifact assemblages (Tiné 1962, 1964). The kinds of human-cave interaction over time were only marginally addressed, and the occupation of the cave was interpreted as secular, related to domestic activities and devoid of any cultic character (Tiné 1964). This reflected the routine application of interpretive models without sufficient data support (Tomkins 2009). In light of advances in the elaboration of theoretical approaches in cave archaeology, we believe that the interpretation of human presence at Grotta di Sant’Angelo III should be reconsidered by future research. For instance, it should be taken into account that the area of the findings was shrouded in darkness, and that the physical conditions of the cave were described by Tiné (1964) himself as highly uncomfortable. Furthermore, other discoveries in the sector known as ‘Grotta di Sant’Angelo II’, revealed types of evidence that various authors have linked with cultic and funerary spheres (Gasparo 1979, 1980; Tiné 1987; Ippolito 2017). This holds in particular for the area at ‘Grotta di Sant’Angelo II’ characterized by significant floor openings, where in the 1970 s pottery dating to the Middle Neolithic I and the Bronze Age was found. Interestingly, in the same cave area human remains were discovered in association with Early Bronze Age artefacts. Previous findings from the Grotta di Sant’Angelo Cave Complex need to be re-examined in light of the insights provided by this study of the pottery from the Trivio area of sector ‘Grotta di Sant’Angelo I’. Pottery is present in all the archaeological sub-contexts of the underground, and its chrono-cultural and functional analysis through the lens of a contextual approach may provide important inferences on the kinds of human engagement with the Grotte di Sant’Angelo Cave Complex and its physicality. Regarding the spatial connections between the various sectors of the underground complex, it should be recalled that sectors ‘Grotta di Sant’Angelo II’ and ‘Grotta di Sant’Angelo III’ form the uppermost level of the Grotte di Sant’Angelo Cave Complex. However, prehistoric people could not reach the level of the Trivio from their chambers and passages – at least not without passing through a 10 m shaft. Logically, they would have accessed the Trivio only through the entrance named ‘Grotta di Sant’Angelo I’ by Tiné (1964), which is now largely obliterated by sediments (Figs. 2a and 3f). Another cave located in the Monte San Marco hill and formed through sulfuric acid speleogenesis – Grotta Pavolella (Gasparo 1979, 1980; Galdenzi 1997) – was also analyzed using a culture-historical approach. This cave opens approximately 200 m northwest of the entrance to the ‘Grotta di Sant’Angelo III’ sector. Archaeological findings there testify to the use of the underground spaces for funerary purposes, with documented rituals during the Middle Neolithic I and the Early Copper Age (Carancini and Guerzoni 1987; Guerzoni 2004; Larocca 2018). Pottery findings are abundant, and most of the Copper Age material from the Trivio area finds comparisons with shapes and decorative styles found at Grotta Pavolella. However, no clear chrono-cultural, spatial, and functional data are available from that site, nor has the evidence been discussed in terms of cave physicality.

## Conclusions

The in-depth chrono-cultural and functional study of ceramic containers from the Trivio area of the Grotte di Sant’Angelo Cave Complex has made it possible to test and evaluate the potential of pottery-based studies when adopting a contextual approach to archaeological caves. This case study demonstrates that the proposed techniques enable scholars working in European cave archaeology to collect a wide range of data appropriate for exploring the six contextual dimensions of relevance identified by recent theoretical advancements. The contributions of the chrono-cultural and functional investigation techniques vary in weight, depending on the contextual dimension being analysed, with each technique offering a more significant contribution to certain aspects while still supporting an overall, integrated assessment.

The determination of the chrono-cultural framework of the pots brought to the cave provides insight into the timeframe of human presence there, as well as the cave’s place with in the broader framework of known sites, patterns of human landscape occupation, circulation of cultural models, and the distribution of material culture (temporal, spatial, and socio-economic contexts).

The functional analysis of ceramic containers allows inferences about the kinds of pots brought to the cave by period and their past contents as part of the general inquiry into the activities conducted at the site and the nature of human-cave entanglements (temporal, ‘architectural’ and socio-economic contexts). Moreover, it helps to investigate how the ceramic containers were brought to their findspots, whether they were specifically fabricated for abandonment in the cave or had first been extensively used, and whether the activities evidenced by use-alteration traces on the pots could comfortably and easily have been performed in dark zones (‘architecture’ context). Defining the types of food resources introduced into the cave makes it possible to discuss their origin from the underground/aboveground realm and to provide insights into foodways as part of the broader investigation of economic strategies and the place of caves within them (spatial and socio-economic contexts).

When combined, the proposed techniques contribute to outlining the history of cave occupation (temporal context) and to speculating on whether the activities conducted in caves involved a major role for abstract thinking (‘architectural’ and socio-economic contexts). In addition, data acquired from chrono-cultural and functional investigations help assess the value of the cave in terms of stratigraphy formation and the preservation of original artefacts, as well as the effects of anthropization on the underground ecosystem and the archaeological depositional context through time (stratigraphy and preservation contexts). A critical discussion of all this information regarding light of the history of archaeological research highlights weaknesses in traditional interpretations of human-cave interactions and suggests new research angles to be applied and tested in the future (scholarly context). Finally, understanding the relationship between the stylistic and functional aspects of pottery contributes to reconstructing past identities and the ritual actions conceived and performed by past populations in relation to both underground and aboveground realms (socio-economic context). As a general consideration on the case study, future research on the Grotte di Sant’Angelo Cave Complex should take into account that the finds suggest a strong relationship between the ritual use of underground spaces – particularly the dark cavities in the cave floor – and the procurement and processing of foodstuffs as part of daily subsistence practices and the means of living of the communities using the cave. Whether this link materialized in the course of secular or ritual activities, during prolonged presences or periodic uses of cave spaces, is difficult to determine. Future research on the relationship between the 4th millennium BCE communities and the Grotte di Sant’Angelo Cave Complex should also consider that such activities do not necessarily fit within the traditional interpretative dichotomy of ‘domestic’ versus ‘ritual’, a distinction whose boundaries are often unclear or shifting, particularly for Prehistoric times.

To conclude, the detailed chrono-cultural and functional study carried out within a contextual approach has revealed its potential for addressing theoretically informed issues. This work thus demonstrates that multidisciplinary pottery analysis is a valuable tool available to scholars working in the field of European cave archaeology.

## Supplementary Information

Below is the link to the electronic supplementary material.

Supplementary Material 1

Supplementary Material 2

## Data Availability

No datasets were generated or analysed during the current study.

## Footnotes

1 DMS geographical coordinates of Grotte di Sant’Angelo Cave Complex: 39°47’19” N 16°18’27” E. These coordinates refers to the leveled area situated in front of the multiple cave entrances and were obtained from the Google Earth platform.

2 The definition of pottery shapes was made following general guidelines by Rice (1987), Cocchi Genick (2008), and Bailo Modesti and Salerno (1998).

3 The pyrolytic degradation products retene and methyl retene are absent from obtained lipid profiles and thus is not possible to interpret betulin and longiborneol presence in samples as markers for pitch production (Whelton et al. 2021).

4 Consumption of horse adipose is eventually suggested by isotopic signature of C16:0 and C18:0 fatty acids only and remains dubious in absence of further confirmation by analysis of faunal remains from the Trivio area.
